# Animal Venoms as Peptide Libraries for the Discovery of Antiglioblastoma Agents

**DOI:** 10.1155/bri/8307315

**Published:** 2026-01-02

**Authors:** Livia Ramos Santiago, Edgar Alejandro Pinos Tamayo, Daniel Moreira dos Santos, Bonglee Kim, José R. Almeida, Rosy Iara Maciel de Azambuja Ribeiro

**Affiliations:** ^1^ Department of Experimental Pathology, Federal University of São João Del-Rey, Divinópolis, Brazil; ^2^ Department of Pathology, College of Korean Medicine, Kyung Hee University, Seoul, Republic of Korea, khu.ac.kr; ^3^ Department of Biomolecules Discovery, Universidad Regional Amazónica Ikiam, Tena, Ecuador; ^4^ School of Pharmacy, University of Reading, Reading, UK, reading.ac.uk

**Keywords:** bioactive peptides, bioinformatic tools, brain tumors, natural sources

## Abstract

Glioblastoma is one of the most aggressive and widely recognized types of brain tumors, characterized by significant cellular and molecular diversity and an inherently aggressive nature. The treatment remains highly challenging, with limited effectiveness and persistently low survival rates. For this reason, researchers are continuously expanding the chemical space of anticancer agents by exploring complex sources such as animal venoms and incorporating in silico tools to accelerate discovery. Indeed, venoms serve as libraries of proteins and peptides, providing a rich source of novel chemical structures for glioblastoma therapy. Some review articles have examined the mechanisms by which venom‐derived peptides target glioblastoma cell lines; nevertheless, key structural insights and computational analyses remain underexplored. In this era of artificial intelligence (AI) and advancements in *in silico* approaches, our review documented the antiglioblastoma properties of venom peptides and underscores the value of computational methods in peptide‐based drug development. To this end, a comprehensive search was conducted in PubMed, Elsevier, Springer, Lilacs, Google Scholar, and SciELO databases. Furthermore, in silico analyses were conducted to evaluate the anticancer potential, hemolytic activity, toxicity, and blood–brain barrier (BBB) penetrating properties of venom‐derived peptides. In total, 26 unique sequences were identified, with their structural properties and mechanisms of cell death comprehensively characterized. The development of peptide‐based anticancer drugs remains in its early stages, with minimal advancement toward preclinical evaluation using in vivo models. The advancement of AI models offers opportunities to accelerate peptide discovery. However, our case study revealed divergences among AI‐based predictions, as well as discrepancies between computational and experimental findings, underscoring the need for further model refinement and validation through experimental data integration. In summary, venoms remain promising peptide libraries that offer valuable natural molecular templates. These peptides require chemical optimization to enhance their stability and BBB permeability. Such advances could enable selective targeting within the glioblastoma niche and support the development of more effective therapies.

## 1. Introduction

Gliomas are one of the types of cancer with the highest mortality rates worldwide and significant economic burden [1]. They are more common in adolescents and children, in addition to being the leading cause of cancer‐related death in men over 40 years and women less than 20 years [1]. Among brain tumors, gliomas stand out as the most prevalent. Primary brain tumors constitute around 81% of all central nervous system neoplasms [2]. Gliomas occur at any age and are more common in adult men [3]. Despite advances in diagnosis and treatment protocols, the prognosis of glioma remains poor. This is especially evident in patients with glioblastoma, the most malignant subtype of glioma, whose median survival remains approximately 15 months despite current therapeutic advances [4].

Gliomas lack a clearly defined origin, but they are often attributed to genetic alterations in brain cells, most likely neural stem cells or progenitor cells [5]. In addition, gliomas represent a heterogeneous group of tumors, classified into five distinct categories based on their histological characteristics and molecular signatures: (1) adult‐type diffuse gliomas; (2) pediatric‐type diffuse low‐grade gliomas; (3) pediatric‐type diffuse high‐grade gliomas; (4) circumscribed astrocytic gliomas; and (5) ependymal tumors [5]. The current therapeutic options for glioblastoma remain limited and fall short of the ideal [6]. They consist of surgical tumor removal, dependent on its location and size, complemented by radiotherapy and the use of the oral alkylating agent temozolomide. Despite advances in treatment, the survival rate is still low, and the associated off‐target effects continue to profoundly impact patients’ quality of life [6].

The identification of novel bioactive agents for developing next‐generation drugs to combat glioblastomas remains an urgent and unresolved task [7, 8]. The journey of glioblastoma therapy can be shaped by multidisciplinary efforts, the implementation of artificial intelligence (AI)–driven discovery technologies, and novel ideas that allow us to circumvent this aggressive and treatment‐resistant cancer [9, 10]. An increasing number of studies have adopted a promising strategy, leveraging the intrinsic ability of animal venom–derived peptides to modulate biological processes in human cells for the discovery of novel drug candidates [11, 12]. This strategy aims to inspire novel and innovative solutions for future glioblastoma therapies [13].

Animal venoms are complex and dynamic biological cocktails of proteins and peptides acting as modulators of different cell processes [14, 15]. They are constantly evolving, creating diverse arrays of toxins with specific functions that can be harnessed to target critical mechanisms in various diseases, including brain tumors [16, 17]. The use of venoms from different animals (snakes, spiders, and scorpions) has been documented in traditional medicine for millennia, addressing a variety of conditions, including arthritis, cancer, and gastrointestinal‐related issues [18]. Most of these treatments have used small doses of crude venoms to achieve their therapeutic effects while minimizing the toxicity [19]. In the 21st century, a distinct approach has emerged, emphasizing the biochemical separation and isolation of individual compounds, followed by detailed investigations of their pharmacological activities [20]. Chromatographic and omics approaches have facilitated the purification and identification of a diverse array of peptide structures with promising therapeutic potential [21, 22]. Recently, advances in bioinformatics and AI algorithms have been accelerating the identification of promising lead candidates [23, 24].

Peptides are recognized as a drug class by the Food and Drug Administration (FDA), with historical contributions for research and therapeutics [25, 26]. They have successfully entered the pharmaceutical market, carving out a promising niche [27]. These short molecules possess several important advantages, such as ease of synthesis, the ability to act through multiple mechanisms of action, and the capacity to bind and penetrate cell membranes [11, 27]. In addition, they do not accumulate in specific organs such as the kidneys or liver, minimizing their adverse effects [28]. More than 30 peptide‐based therapeutic agents targeting glioblastoma have progressed into clinical translation, most of which are currently or have been evaluated in Phase I and Phase II trials [29]. Notably, the majority of the candidates are peptide‐based vaccines, reflecting the strong emphasis on immunotherapeutic strategies against glioblastoma. However, cell‐penetrating peptides (CPPs) are also represented among the candidates, acting through direct cytotoxic mechanisms or by interfering with tumor cell division [29]. Together, these approaches underscore the versatility of peptide‐based drugs in oncology, capable of eliciting immune‐mediated tumor suppression while also exerting direct antineoplastic activity [30].

Venom‐derived peptides have also shown remarkable progress along the translational pathway, leading to the development of several clinically available or late‐stage therapeutic agents [11]. There are currently nine drugs approved by the FDA derived from animal venoms: captopril (snake, hypertension), enalapril (snake, hypertension), ziconotide (cone snail, chronic pain), lepirudin (medicinal leech, stroke, deep vein thrombosis, and pulmonary embolism), bivalirudin (medicinal leech, coagulation during surgery), batroxobin (snake, perioperative bleeding), apitox (bee, osteoarthritis), cobratid (snake, pain), and exenatide (lizard, Type 2 diabetes) [20, 31]. In the field of oncology, peptides derived from venom libraries remain in the early phases of development. Chlorotoxin (*Leiurus quinquestriatus*—glioblastoma), crotoxin (*Crotalus durissus terrificus*—solid tumors), and BLZ‐100 (*Leiurus quinquestriatus quinquestriatus*—skin cancer and glioma) have progressed to clinical trials but have not yet reached the market [32, 33].

This review focuses on animal venom‐derived peptides targeting glioblastoma cells. We compiled valuable information on their sequences, mechanisms of action, sources, and chemical modifications. By employing freely available AI‐based tools, we also showcase the role and challenges of computational approaches in the discovery and validation of novel potential antiglioblastoma peptides. In summary, we integrated in silico, in vitro, and in vivo perspectives to provide a better picture of the progress, advances, and remaining obstacles in the development of venom‐derived peptides for glioblastoma therapy.

## 2. Methods

A search across PubMed, Elsevier, Springer, and SciELO was conducted to identify studies on animal venom‐derived peptides exhibiting antitumor activity over glioblastoma cell lines and/or in vivo models. Descriptors were grouped (indexed in DeCS Integrated Advanced Information Management Systems Trillian Health Sciences Descriptors) and Medical Subject of Health (MeSH) terms as follows: brain tumors, glioblastoma, glioma, peptide venom, and venom. The inclusion criteria were as follows: (1) publications written in English, Portuguese, or Spanish and published between January 1990 and August 2024; and (2) studies conducted with animal venom‐derived peptides toxicity in vitro and/or in vivo in brain tumors. Studies that did not examine the effects of these molecules on glioblastoma cell lines were excluded. The information collected and summarized in this work includes the animal species, name of the peptide, number of amino acids, primary structure, cell lines, concentrations, effective dose, treatment time, and the mechanism(s) of action. The initial search gave 12,766 articles. According to the established inclusion criteria, 12,738 articles were excluded. Finally, a total of 26 articles were included in the development of this work.

### 2.1. A Computational View on the Hemolytic, Cytotoxic, Anticancer, and Blood–Brain Barrier (BBB) Penetrating Properties of Venom‐Derived Peptides

Different open access tools were used to predict the hemolytic activity and toxicity of the different peptides derived from animal venoms. In this study, the predictors HemoPi2 (https://webs.iiitd.edu.in/raghava/hemopi2/) [34] and HLPpred‐Fuse (http://thegleelab.org/HLPpred-Fuse/) [35] were used to estimate hemolytic activity, and ToxinPred 3.0 (http://crdd.osdd.net/raghava/toxinpred/) [36], CSM toxicity (https://biosig.lab.uq.edu.au/csm_toxin/) [37], and pkCSM (https://biosig.lab.uq.edu.au/pkcsm/) [38] were used to assess the overall toxicity of the analyzed peptides. To highlight the value of AI in peptide‐based oncology research, we conducted a comprehensive reassessment of antiglioblastoma peptides with documented in vitro or in vivo effects, using state‐of‐the‐art AI‐based prediction platforms to estimate their anticancer potential. The tools used were AntiCP 2.0 (https://webs.iiitd.edu.in/raghava/anticp2/) [39] and MLACP 2.0 (https://balalab-skku.org/mlacp2/) [40]. Furthermore, the ability of peptides to penetrate the BBB was assessed using two computational webservers: BBBper (https://bbbper.mdu.ac.in/) and LogBB_Pred (http://ssbio.cau.ac.kr/software/logbb_pred/) were used [41, 42].

## 3. The Landscape of Animal Venom–Derived Peptides in Glioblastoma

There are over 540,000 known venomous species worldwide, each serving a rich library of peptides with potential therapeutic applications. Of all these peptides, approximately 7000 have been identified, representing less than 0.02% of the total biochemical space [43]. In our analysis, we identified 26 peptides with in vivo or in vitro action against glioblastoma cells from different sources. Table 1 provides an overview of the primary structures, amino acid lengths, methods of obtention, and structural modifications of antiglioblastoma peptides.

**Table 1:** Primary structures of animal venom–derived peptides with activity against different glioblastoma cell lines.

Animal group	Peptide name	Sequences	Length (aa)	Obtention method	Modifications
Arachnids	Psalmotoxin 1	EDCIPKWKGCVNRHGDCCEGLECWKRRRSFEVCVPKTPKT	40	Natural	No modifications
	Phα1β	ACIPRGEICTDDCECCGCDNQCYCPPGSSLGIFKCSCAHANKYFCNRKKEKCKKA	55	Natural	No modifications
	PhTx3‐3	GCANAYKSCNGPHTCCWGYNGYKKACICSGcolor = “#FF0000;”>XNWK	34	Natural	No modifications
	LyeTxI‐b	IWLTALKFLGKNLGKLAKQQLAKL	24	Synthetic	Acetylation at the N‐terminus and amidation at the C‐terminus
	Chlorotoxin	MCMPCFTTDHQMARKCDDCCGGKGRGKCYGPQCLCR	36	Synthetic	Biotinylated at the N‐terminus
	BmKCT	MKFLYGIVFIALFLTVMFATQTDGCGPCFTTDANMARKCRECCGGIGKCFGPQCLCNRI	59	Recombinant	No modifications
	AaCTx	MCIPCFTTNPNMAAKCNACCGSRRGSCRGPQCIC	34	Natural and synthetic	No modifications
	Acra3	MKIIFLVLMMILSEVYSDRDGYPVHDGTNCKYSCDIREKWEYCTPLCKRRNAKTGYCYAFACWCIGLPDEVKVYGDDGIFCKSG	84	Natural	No modifications
	KAaH1	ADVPGNYPLDSSDDTYLCAPLGENPFCIKICRKHGVKYGYCYAFQCWCEYLEDKNVKI	58	Natural	No modifications
	KAaH2	ADVPGNYPLDSSDDTYLCAPLGENPSCIQICRKHGVKYGYCYAFQCWCEYLEDKNVKI	58	Natural	No modifications
	RK	IDCGTVMIPSECDPKSS	17	Synthetic	No modifications
	RK1	IDCSKVNLTAECSS	14	Synthetic	No modifications
	AaTs‐1	LWSK	4	Natural and synthetic	No modifications
	P01	VSCEDCPEHCSTQKAQAKCDNDKCVCEPI	29	Natural	No modifications
	Androcin 18−1	IFGTVFKLFKFIPGIAKLFKKKKE	24	Synthetic	No modifications

Insects	Anoplin	GLLKRIKTLL	10	Synthetic	Amidation at the C‐terminus
	MP1	IDWKKLLDAAKQIL	14	Synthetic	Amidation at the C‐terminus
	MPX	INWKGIAAMAKKLL	14	Synthetic	Amidation at the C‐terminus
	HR1	INLKAIAALVKKVL	14	Synthetic	Amidation at the C‐terminus
	Melittin	GIGAILKVLATGLPTLISWIKNKRKQ	26	Synthetic	No modifications
	Apamin	CNCKAPETALCARRCQQH	18	Natural and synthetic	No modifications

Ophidians	PIVL	color = “#FF0000;”>ZDRPKFCYLPADPAECNAYMPRFYYDSASNKCKEFIYGGCRGNANNFKNRAECRHTCVASRKGIQPR	67	Natural	No modifications
	Contortrostatin	DAPANPCCDAATCKLTTGSQCADGLCCDQCKFMKEGTVCRRARGDDLDDYCNGISAGCPRNPFH	64	Natural	No modifications
	Vicrostatin	GDAPANPCCDAATCKLTTGSQCADGLCCDQCKFMKEGTVCRRARGDDLDDYCNGISAGCPRNPHKGPAT	69	Synthetic	No modifications
	Myotoxin‐3	YKRCHKKGGHCFPKTVICLPPSSDFGKMDCRWKWKCCKKGSVNNA	45	Synthetic	Folded/oxidized using a Tris‐ACN solution, after the synthesis
	VLO4	MNSGNPCCDPVTCKPRRGEHCVSGPCCRNCKFLNAGTICKRARGDDMNDYCTGISPDCPPRNPWKG	66	Natural	No modifications

*Note:* Length and structural modifications are also included. Residues *X* and *Z* (highlighted in red) denote the unidentified or ambiguous amino acids.

Among the peptides analyzed, arachnid‐derived molecules constitute the most represented group, exhibiting significant diversity in sequence length, charge, and structural complexity. Their sequences range from very short peptides such as AaTs‐1 (4‐mer) and LyeTxI‐b (24‐mer) to longer cysteine‐rich toxins like Phα1β (55‐mer) and Acra3 (84‐mer). Most arachnid peptides are cationic, enriched in lysine and arginine residues, which can facilitate electrostatic interactions with negatively charged cell membranes, an attribute commonly linked to membrane‐disruptive effects or ion‐channel activity. Several of these peptides, such as chlorotoxin, are synthetically produced or biotinylated to enhance targeting and imaging capabilities, while others remain naturally isolated with no chemical modifications, preserving their native disulfide‐bridged architectures.

In contrast, insect‐derived peptides, including anoplin, MP1, MPX, HR1, melittin, and apamin, are generally short (10–26 aa), amphipathic, and highly cationic, favoring direct interaction with lipid bilayers. These peptides are mostly synthetic and frequently C‐terminally amidated, a chemical modification that enhances structural stability and membrane affinity. Their simple linear structures and positive charge distribution make them valuable candidates for structure–activity optimization and AI‐guided drug design aimed at improving selectivity and reducing cytotoxicity.

Meanwhile, snake venom–derived peptides, such as contortrostatin, vicrostatin, Myotoxin‐3, and VLO4, are typically longer (45–69 aa) and disulfide‐rich, reflecting a high degree of structural organization. Most are naturally derived, with minimal or no chemical modification, although synthetic analogs like vicrostatin have been developed to improve stability and integrin‐binding properties. These peptides often exhibit anti‐adhesive and anti‐invasive activities, targeting integrins and extracellular matrix components rather than directly disrupting cell membranes.

Overall, this table reveals trends between the source, length, and structural modification. Natural peptides tend to be longer and stabilized by disulfide bonds, synthetic peptides are shorter and often chemically optimized through amidation or labeling, and only a few recombinant variants have been reported. This balance between natural molecular complexity and synthetic refinement underscores the versatility of venom‐derived peptides as attractive scaffolds for rational drug design and antiglioblastoma therapy development.

The analysis also revealed that the rational engineering of venom‐derived peptides has remained limited, particularly when compared to the rapid advancements in modern peptide chemistry. To date, most modification efforts have primarily relied on C‐terminal amidation, a common strategy employed to enhance peptide stability and resist enzymatic degradation. However, this represents only a fraction of the available chemical space for optimization. Other approaches, such as N‐terminal acetylation, cyclization, incorporation of non‐natural amino acids, PEGylation, or lipidation, could be employed to further improve pharmacokinetic properties, membrane permeability, target selectivity, and in vivo stability [44, 45]. Expanding beyond conventional amidation‐based modifications may therefore unlock the full therapeutic potential of venom‐derived peptides, enabling the development of more potent and selective antiglioblastoma agents.

Analysis of the peptide net charges revealed physicochemical trends related to antiglioblastoma potential. Most peptides displayed net positive charges, typically ranging from +3 to +9, indicating a high content of lysine (K) and arginine (R) residues. This cationic character is usually reported in the literature as crucial for interactions with negatively charged components of glioblastoma cell membranes, such as phosphatidylserine and glycosaminoglycans [29]. In contrast, a smaller subset of peptides showed neutral or slightly negative net charges (0 to −3). These are typically larger, disulfide‐rich molecules with constrained structures, suggesting alternative mechanisms of action beyond direct membrane disruption, such as antiangiogenic or receptor‐mediated effects. This duality between highly cationic and near‐neutral peptides reflects the diversity of venom‐derived molecules, combining electrostatic‐driven cytotoxicity with specific molecular targeting. Overall, the predominance of positively charged peptides highlights their potential for glioblastoma therapy, although fine‐tuning of charge balance remains essential to optimize selectivity and reduce toxicity to normal brain cells.

**Figure 1:**
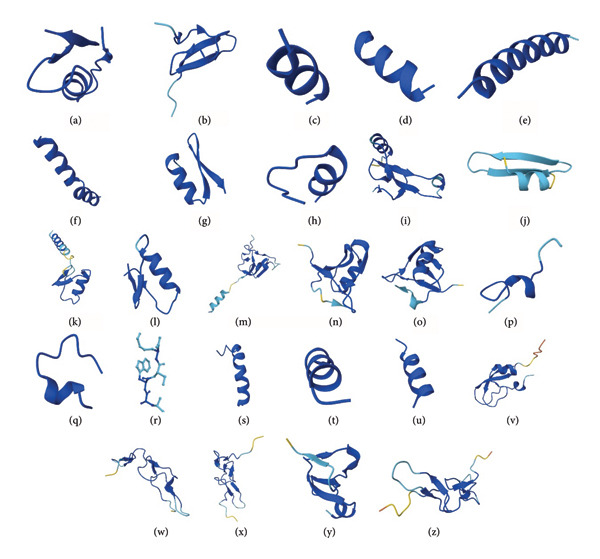
Diversity of the three‐dimensional structures of venom‐derived peptides active against glioblastoma. (a) Chlorotoxin; (b) psalmotoxin; (c) anoplin; (d) Mastoparan‐X; (e) LyeTx‐Ib; (f) melittin (PDB: 2MLT); (g) P01; (h) apamin; (i) Phα1β; (j) PhTx3‐3; (k) BmKCT; (l) AaCTx; (m) Acra3; (n) KAaH1; (o) KAaH2; (p) RK; (q) RK1; (r) AaTs‐1; (s) Androcin 18−1; (t) MP1; (u) HR1; (v) PIVL; (w) contortrostatin; (x) vicrostatin; (y) Myotoxin‐3; (z) VLO4. Structural models were predicted using the AlphaFold Server.

At the structural level, antiglioblastoma peptides derived from venoms exhibit intriguing diversity. Figure 1 illustrates this structural heterogeneity, showcasing the wide range of conformations these bioactive molecules can adopt. Each subfigure (a–z) represents a distinct three‐dimensional fold, encompassing α‐helices, β‐sheets, random coils, and mixed secondary‐structure motifs. Among these, α‐helical conformations predominate, reflecting their well‐established role in facilitating membrane interaction and cellular uptake, processes that are fundamental to anticancer mechanisms. This structural variability underpins the functional versatility of antiglioblastoma peptides, enabling them to interact with a broad spectrum of molecular targets implicated in glioblastoma progression, including membrane components, intracellular targets, and signaling proteins. The diversity in folding patterns, ranging from compact helical bundles to extended β‐sheet frameworks, suggests the peptides’ distinct mechanisms of action that may be exploited for the development of innovative glioblastoma therapies.

At the mechanistic level, venom‐derived peptides exhibit multifaceted properties and employ a diverse range of mechanisms, including the inhibition of proliferation, the induction of apoptosis, necrosis, and/or necroptosis; and the inhibition of angiogenesis, invasion, and/or metastasis [46]. Historically, the mode of action of venom‐derived peptides has been elucidated by combining different approaches, which involve experimental validation combined with computational methods that can facilitate experimental validation [47]. In the following sections, we examine these venom‐derived peptides in detail to provide a comprehensive overview of their potential of in glioblastoma drug discovery and development.

### 3.1. Spider and Scorpion Venoms Are the Most Studied Sources of Glioblastoma‐Targeting Peptides Among Arachnids

#### 3.1.1. Spider Venom–Derived Peptides Tackling Glioblastoma

Among all venom‐producing animals, spiders comprise 50,033 described species [48]. Spider venom is rich in proteins and peptides with antimicrobial, neurotoxic, analgesic, antitumor, and necrotic activities [49]. Omics approaches and their combinations have been used to gain more information about the components present in spider venom [50]. Venom‐mining investigations have uncovered the biochemical diversity and richness that characterize these complex natural cocktails [51]. Some of these molecules have been shown to target several distinct ion channels and receptors [52, 53]. However, spider venoms remain largely underexplored, positioning them as promising resources for bioprospecting applications [54]. The literature is heavily focused on the in vitro potential of venom‐derived peptides.

**Figure 2:**
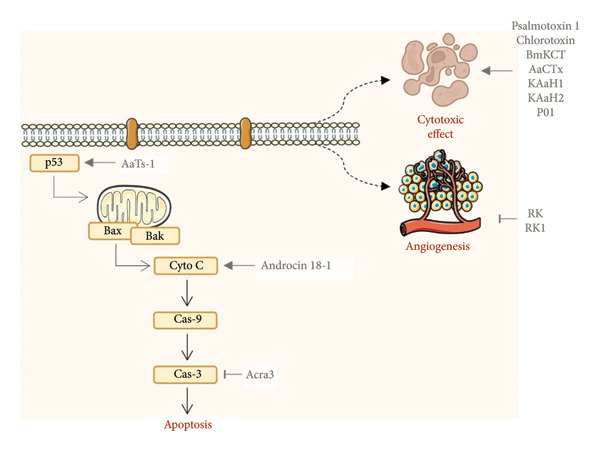
Venom‐derived peptides from spiders and scorpions modulate molecular events in glioblastoma cells in vitro. Abbreviations: ↓, upregulation; T inhibit; p53: tumor protein p53; Bax, Bcl‐2–associated X protein; Bak: Bcl‐2 homologues antagonist/killer; Cyto C: Cytochrome C; Cas‐9: Caspase‐9; Cas‐3: Caspase‐3. Image provided by Servier Medical Art (https://smart.servier.com/), licensed under CC BY 4.0 (https://creativecommons.org/licenses/by/4.0/).

Table 2 summarizes the experimental parameters and biological responses of spider venom–derived peptides tested in vitro against glioblastoma cell lines, highlighting their cytotoxic and channel‐modulating activities. Although arachnid‐derived venom peptides are among the most extensively studied, only four spider peptides have been tested in vitro against glioblastoma. These include Psalmotoxin 1 (PcTx1), evaluated in SK‐MG‐1, U251‐MG, U87‐MG, and D54‐MG cell lines; Phα1β, tested in M059J, U138‐MG, and U251‐MG; and LyeTxI‐b, assessed in U87‐MG cells. The mechanisms of action and molecular targets of these antiglioblastoma peptides are illustrated in Figure 2.

**Table 2:** In vitro action of spider venom– and scorpion venom–derived peptides against glioblastoma cell lines.

Source (type of animal)	Peptide names (aa number)	Tumors cell line	Nontumor cell line	Dose	IC_50_	Time (h)	Outcome	Toxicity assay	Ref.
*Psalmopoeus cambridgei* (spider)	Psalmotoxin 1 (40)	SK‐MG‐1; U251‐MG; U87‐MG	—	1 nM	36 pM	NR	Cytotoxic effect	—	[55]
*Psalmopoeus cambridgei* (spider)	Psalmotoxin 1 (40)	D54‐MG	—	100 nM	—	5	Cytotoxic effect	—	[56]
*Phoneutria nigriventer* (spider)	Phα1β (55)	M059J; U‐138MG; U‐251MG	N9	10 pM	—	18, 24	Necroptosis	—	[57]
*Lycosa erythrognatha* (spider)	LyeTxI‐b (24)	U87	Vero, PBMC, GM637, hRBC	29.20 µM		48	Necroptosis	Hemolytic activity (low)	[58]
*Leiurus quinquestriatus* (scorpion)	Chlorotoxin (36)	U373MG; U105MG; U251MG	—	15 µCi	—	24, 48, 72, 96	Cytotoxic effect	—	[59]
*Buthus martensii Karsch* (scorpion)	BmKCT (59)	C6	—	0.15, 0.30, 0.6, 1.2, and 2.4 µM	—	24	Cytotoxic effect	—	[60]
*Androctonus australis* (scorpion)	AaCTx (34)	U87	—	10 µM		48	Cytotoxic effect	In vivo toxicity test	[61]
*Androctonus crassicauda* (scorpion)	Acra3 (84)	BC3H1	F2408, 5RP7, NIH3T3	5.5 and 3 μg/mL	5.5 μg/mL	24 and 48	Necrosis ↓ Caspases 3 and 6	—	[62]
*Androctonus australis* (scorpion)	AaCTx (34)	*In silico*					Cytotoxic effect	—	[63]
*Androctonus australis Hector* (scorpion)	KAaH1 (58)	U87	—	50 μg/mL		72	Cytotoxic effect	—	[64]
*Androctonus australis Hector* (scorpion)	KAaH2 (58)	U87	—	50 μg/mL		72	Cytotoxic effect	—	
*Buthus occitanus tunetanus* (scorpion)	RK (17)	U87	Human fibrinogen	100 μM	15 µM	72	Antiangiogenic	In vivo toxicity test (not exhibit any toxicity)	[65]
*Buthus occitanus tunetanus* (scorpion)	RK1 (14)	U87	—	2 and 4 µM	—	24	Antiangiogenic	In vivo toxicity test (not exhibit any toxicity)	[66]
*Androctonus australis* (scorpion)	AaTs‐1 (4)	U87	—	0.56 mM		24, 48, 72 ↑ p53 ↓ ERK, p38, JNK	Apoptosis	In vivo toxicity test (not induce mice mortality)	[67]
*Androctonus australis* (scorpion)	P01 (29)	U87	—	25–200 μg/mL	10 μg/mL	24, 48, 72	Cytotoxic effect	—	[68]
*Androctonus bicolor* (scorpion)	Androcin 18−1 (24)	U87	—	0.845 μM		24	Apoptosis ↑ Cytochrome c and Caspase 3	—	[69]

*Note:* This table summarizes studies that evaluated the antitumor effects of venom‐derived peptides in in vivo models of glioblastoma. Information includes the animal of origin, the name and length (number of amino acids) of the peptide, the brain tumor cell line, the dose administered, the duration of treatment, the animal model, and the route of administration. Data were compiled from the cited references.

Abbreviation: aa, amino acids.

Initial studies with LyeTx I‐b, a synthetic and modified 24‐mer peptide inspired by LyeTx from the venom of *Lycosa erythrognatha*, demonstrated potent antimicrobial activity against both Gram‐positive and Gram‐negative bacteria [70]. By repurposing strategy, LyeTx I‐b demonstrated cytotoxic effects on glioblastoma cell lines, primarily inducing necroptosis [58]. The venom of *Phoneutria nigriventer*, a species found in the forests of Central and South America, particularly from the eastern Andes to northern Argentina, has been extensively studied due to its medical importance [71]. In *in vivo* and in vitro assays, the isolated peptide Phα1β (55‐mer) from this venom was shown to inhibit the proliferation and viability of glioblastoma cell lines (U‐138MG and U‐251MG). This peptide also promoted morphological changes consistent with apoptosis in U‐251MG and necrosis in U‐138MG [57]. Furthermore, Psalmotoxin 1, a 40‐mer peptide isolated from the spider *Psalmopoeus cambridgei*, exhibited promising cytotoxic and antiangiogenic activities in different glioblastoma cell lines, including SK‐MG‐1, U251‐MG, U87‐MG, and D54‐MG [55, 56]. Although limited examples exist, some spider venom–derived peptides have demonstrated in vivo potential (Table 3).

**Table 3:** In vivo action of scorpion venom– and spider venom–derived peptides against glioblastoma.

Animal	Peptide names and aa number	Brain tumors cell line	Dose	Time	Animal model	Route of administration	Outcome	Ref.
*Phoneutria nigriventer* (spider)	Phα1β (55)	GL261	50 pmol/site	20 days	Male C57/BL6 wild‐type orthotopic mouse	Intracerebroventricular	Reduces glioma tumor growth	[57]
*Phoneutria nigriventer* (spider)	PhTx3‐3 (34)	GL261	50 pmol/site	20 days				

*Buthus occitanus tunetanus* (scorpion)	RK1 (14)	U87	2, 4, and 8 µM	48 h	Chicken embryo	Topical application	Inhibition of angiogenesis	[66]

*Leiurus quinquestriatus* (scorpion)	Chlorotoxin (36)	D‐54MG	50 piCi/mouse	24, 48, 72, 96 h	SCID mice	Intraperitoneal	Cytotoxicity on tumor cells and diagnostics	[72]

*Note:* This table summarizes studies that evaluated the antitumor effects of venom‐derived peptides in in vivo models of glioblastoma. Information includes the animal of origin, the name and length (number of amino acids) of the peptide, the brain tumor cell line, the dose administered, the duration of treatment, the animal model, and the route of administration. Data were compiled from the cited references.

Abbreviations: aa, amino acids.

#### 3.1.2. Scorpion Venom–Derived Peptides With Antiglioblastoma Properties

Scorpions are predatory arachnids that occupy almost all terrestrial habitats, except Antarctica [73]. The study of scorpion venoms was popularized in the 1950s when their protein components were identified for the first time. Similarly to spider venoms, scorpion venoms are considered to be a rich source of pharmacologically active compounds. Their isolated molecules have shown antibacterial, antitumor, antifungal, antiviral, antiparasitic, immunosuppressive, and analgesic activities, making them promising for the development of treatments for various diseases [74].

In Table 2, 11 peptides are identified as originating from scorpion venoms, mainly from species such *as L. quinquestriatus, Androctonus australis, An. crassicauda,* and *Buthus martensii Karsch*. These peptides vary widely in size, from short linear sequences like AaTs‐1 (4‐mer) and RK1 (14‐mer) to longer, disulfide‐rich toxins such as chlorotoxin (36‐mer) and Acra3 (84‐mer), reflecting the structural diversity typical of scorpion venom libraries. Most have been evaluated against the U87‐MG glioblastoma cell line, although some, including chlorotoxin and BmKCT, were also tested in U251‐MG, U373‐MG, or C6 cells. Several peptides, particularly chlorotoxin, AaTs‐1, and RK, demonstrated selective cytotoxicity and inhibition of migration or angiogenesis, with minimal toxicity to nontumor cells.

The diverse mechanisms of action of these peptides encompass matrix metalloproteinase inhibition, the induction of apoptosis or necrosis, and antiangiogenic activity. Figure 2 summarizes the key cellular events modulated by these peptides. Collectively, the findings presented in Table 2 and Figure 2 highlight the therapeutic potential of scorpion venom–derived peptides as selective and multifunctional molecules with promise as lead compounds for glioblastoma drug development. Below, we describe several representative examples in greater detail.

Some isolated compounds and the crude venom from *L. quinquestriatus* have been broadly studied by Purali and Yagcioglu [75]. Among all these compounds, chlorotoxin (36‐mer peptide) has been shown to inhibit the proliferation of several glioblastoma cell lines (D‐54MG, U373MG, U105MG, and U251MG). The radiolabeled chlorotoxin used in the in vivo experiment accumulated in tumors, suggesting its potential for glioblastoma diagnosis [59, 72]. On the other hand, the BmKCT peptide (59‐mer), similar to chlorotoxin isolated from the venom of *B. martensii* Karsch scorpion, was shown to block chloride ion channels and inhibit Matrix Metalloproteinase 2 [60]. The same action can be observed with the AaCTx peptide (34‐mer) in glioblastoma cells [63]. Furthermore, in vitro AaCtx inhibited the migration and invasion of glioblastoma cells [61].

Another well‐studied venom is the scorpion *Androctonus australis*, mainly found in Algeria [76]. AaTs‐1 (4‐mer), a peptide derived from this species, inhibited glioblastoma cell proliferation, increased p53 expression, and inhibited the phosphorylation of the kinases ERK, p38, and JNK, which increases the chemosensitivity of glioblastoma cells [67]. Other isolated peptides, including KAaH1 (58‐mer) and KAaH2 (58‐mer), have inhibited the migration and adhesion of different tumor cell lines, including the glioblastoma cell line U87 [64]. Another peptide isolated from the venom of this scorpion venom, named P01 (29‐mer), inhibited the proliferation, adhesion, and migration of U87. Its activity was associated with the potential of P01 to specifically block the potassium and Calcium Channel 2 (SK2) [68]. Meanwhile, the Acra3 peptide (84‐mer) isolated from *An. crassicauda* inhibited the proliferation of glioblastoma (BH3H1) tumor cells and induced cell death by necroptosis [62].

*B. occitanus tunetanus* is one of Tunisia’s main scorpions involved in human envenomation [77]. Its venom presents the bioactive compounds that have been frequently studied. RK (17‐amino acid) and RK1 (14‐mer) peptides have shown the inhibition capacity of cell adhesion in different tumor cell lines, such as melanoma and glioblastoma (U87) [65, 66]. Additionally, RK1 inhibited migration and angiogenesis [66]. The peptide Androcin 18‐1 (24‐mer) inhibited migration by expressing MMPs/TIMPs, resulting in apoptosis [69].

Although reports remain scarce, some scorpion venom–derived peptides exhibit notable in vivo efficacy (Table 3). The most extensively studied peptide, chlorotoxin (36‐mer) from *L. quinquestriatus*, showed significant antitumor activity in SCID mice bearing D‐54MG glioma xenografts following intraperitoneal administration. Similarly, the short peptide RK1 (14‐mer) from *B. occitanus tunetanus* exhibited antiangiogenic effects in a chicken embryo model, where topical application at micromolar concentrations (2–8 μM) effectively inhibited neovascularization.

### 3.2. Insect Venom: A Source of Peptides With Glioblastoma‐Fighting Activity

Clinically relevant insect venoms include several species of bees, wasps, and ants. These insects’ venoms are complex mixtures of bioactive molecules, small organic compounds, peptides, and proteins. Their main actions include membrane disruption and induction of hypersensitivity reactions [78]. The therapeutic potential of peptides derived from insect venom, particularly bees and wasps, has shown promising results for advancing cancer treatment [79].

In Table 4, five peptides derived from insect venoms, primarily from wasps and bees, are reported to exert cytotoxic effects on glioblastoma cell lines. These include anoplin (10‐mer) from *Anoplius samariensis*, MP1 (14‐mer) from *Polybia paulista*, MPX (14‐mer) from *Vespa xanthoptera*, HR1 (14‐mer) from *V. orientalis*, and melittin (26‐mer) from *Apis mellifera*. The T98G glioblastoma cell line was the common target for most wasp peptides, while melittin was tested against multiple lines, including Hs683, T98G, U373, and DBTRG‐05MG, confirming its broad cytotoxic profile.

**Table 4:** Venom‐derived peptides from insects are toxic to glioblastoma cells.

Source (type of animal)	Peptide names (aa number)	Brain tumors cell line	Dose	Time (h)	Outcome	Ref.
*Anoplius samariensis* (wasp)	Anoplin (10)	T98G	20 μmol L^−1^	2	Mast cell degranulation	[80]
*Polybia paulista* (wasp)	MP1 (14)	T98G	32.70 μmol L^−1^	2	Necrosis	
*Vespa xanthoptera* (wasp)	MPX (14)	T98G	18.05 μmol L^−1^	2	Necrosis	
*Vespa orientalis* (wasp)	HR1 (14)	T98G	12.52 μmol L^−1^	2	Necrosis	
*Apis Mellifera* (bee)	Melittin (26)	Hs683; T98G; U373	7.77 μg/mL (Hs683) 31.53 μg/mL (TG98) 12.34 μg/mL (U373)	72	Apoptosis ↑ Bak e VDAC	[81]
*Apis Mellifera* (bee)	Melittin (26)	DBTRG‐05 MG	2.5 μg/mL	24	Apoptosis ↑ Caspases 3, 8, and 9	[82]

*Note:* This table presents the in vitro evaluation of wasp and bee venom–derived peptides with activity against glioblastoma. Listed data include the source species, peptide name and amino acid length, glioblastoma cell lines tested, applied dose, treatment duration, and the observed biological responses. Data were compiled from the cited references.

Abbreviations: aa, amino acids.

Wasps are distributed throughout the world and comprise more than 5000 species. Wasp venom has many biochemical constituents (e.g., proteins, peptides, enzymes, and other small molecules). Its compounds isolated have demonstrated antimicrobial, antitumor, and anti‐inflammatory activities [83]. Among peptides isolated and identified from these venoms, anoplin (*A. samariensis*) (10‐amino acid), MP1 (*P. paulista*) (14‐mer), MPX (*V. xanthoptera*) (14‐mer), and HR1 (*V. orientalis)* (14‐mer) induced cell death in T98G glioblastoma cell lines mainly by necrosis [80].

Another venom that has been extensively researched and shows promising results is bee venom. Bee venom represents a complex mixture of substances designed to protect bees against predators [84]. Apitherapy is based on the use of bee products, mainly bee venom for the treatment of different diseases such as cancer [85], inflammation, central nervous system diseases such as Parkinson’s disease, amyotrophic lateral sclerosis [86], Alzheimer’s disease [87], and cardiovascular diseases [88]. Among bees, the most studied species is *Ap. mellifera*, which has successfully colonized various ecosystems worldwide [89]. Its venom inhibited the growth of colon cancer cells via apoptosis, with activation of DR4 and DR5 and inhibition of the NF‐κB signaling pathway [90]. In addition, this venom exerted cytotoxic activity and reduced the secretion of MMP‐2 and MMP‐9 from glioblastoma cells [91].

Among the peptides isolated from this venom, melittin (26‐mer) is one of the most well‐studied. It increases Bax and Bak expressions, thus inducing cell death via the intrinsic apoptotic pathway in glioblastoma cell lines (Hs683, T98G, and U373) [81]. In addition, melittin‐sensitized cancer cells to cisplatin treatment in glioblastoma cells (DBTRG‐05MG), activating a series of pathways including an increase in MitSOX production, an accumulation of cytCa^2+^, and a decrease in MitPOT, inducing apoptosis [82]. Another peptide isolated from the same venom, apamin, specifically blocked a class of Ca^(2+)^ K^+^ channels in glioma cell lines [92].

**Figure 3:**
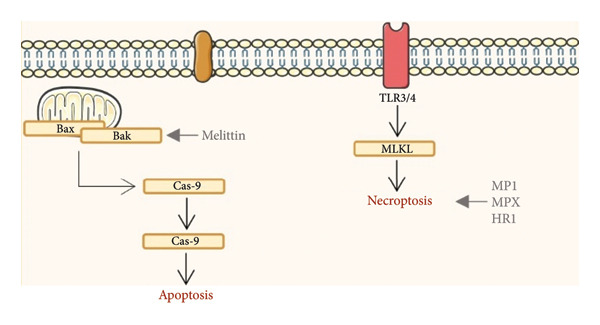
Venom‐derived peptides from wasp and bee modulate molecular events in glioblastoma cells in vitro. Abbreviations: ↓, upregulation; T inhibit; p53: tumor protein p53; Bax, Bcl‐2–associated X protein; Bak: Bcl‐2 homologues antagonist/killer; Cas‐9: Caspase‐9; Cas‐3: Caspase‐3; MLKL: mixed lineage kinase domain–like pseudokinase. Image provided by Servier Medical Art (https://smart.servier.com/), licensed under CC BY 4.0 (https://creativecommons.org/licenses/by/4.0/).

The mechanistic pathways through which insect venom–derived peptides exert cytotoxic effects on glioblastoma cells are illustrated in Figure 3. Melittin promotes apoptosis through mitochondrial activation and caspase signaling, while MP1, MPX, and HR1 induce necroptosis via membrane receptor–mediated MLKL activation, highlighting distinct but complementary death pathways in glioblastoma cells.

### 3.3. Snake Venoms Are Toxic Cocktails With Peptides Selectively Killing Glioblastoma Cells

The use of snake venoms has been documented since ancient times as a biological weapon in battles [93]. Its use in medicine began in the seventh century in Ayurveda to treat gastrointestinal disease, and arthritis, and to promote longevity [93]. Snake venoms are complex mixtures of fast‐acting proteins and peptides that target a wide range of molecular targets and cellular events with physiological implications [94]. With this in mind, snake venom components are the startpoint for the drug development of candidates for different diseases [95]. Captopril® is the first venom‐inspired medicine approved by the FDA. The journey of design and development is based on the discovery of peptides in the Brazilian *Bothrops jararaca* snake venom [96]. This peptide signifies a transformative era in the scientific understanding and clinical management of hypertension. In the same context, snake venoms have been explored as the source of antiglioblastoma agents [16, 17].

Table 5 summarizes seven peptides derived from snake venoms that have demonstrated in vitro activity against glioblastoma or brain tumor cell lines. These peptides include contortrostatin (65‐mer) from *Agkistrodon contortrix contortrix*, PIVL (67‐mer) from *Macrovipera lebetina trasmediterranea*, vicrostatin (69‐mer) derived from *A. contortrix* and *Echis carinatus*, Myotoxin‐3 (45‐mer) from *C. oreganus*, β‐micrustoxin (PLA_2_) from *Micrurus lemniscatus*, and VLO4 (66‐mer) from *Vipera lebetina obtusa*. These peptides were mainly evaluated in U87, U251, U138, and A172 glioblastoma cell lines, using treatment durations ranging from 2 to 24 h, and in some cases extending to 14 days. In continuation, we describe in more detail some of these studies to illustrate the mechanisms and biological effects of snake venom–derived peptides on glioblastoma cells.

**Table 5:** In vitro antiglioblastoma activity of snake venom–derived peptides.

Source (type of animal)	Peptide names (aa number)	Brain tumors cell line	Dose used	Time	Efficacy	Ref.
*Agkistrodon contortrix contortrix* (snake)	Contortrostatin (65)	U87	40 µg	14 days	Antiangiogenic	[97]
*Agkistrodon contortrix contortrix* (snake)	Contortrostatin (65)	T98G; U87; A172; U138	NR	12 h	Antiangiogenic	[98]
*Macrovipera lebetina trasmediterranea* (snake)	PIVL (67)	U87	1 µM	2 h	Antiangiogenic	[99]
*Agkistrodon contortrix* and *Echis carinatus* (snake)	Vicrostatin (69)	U87; U251	100 μCi	18 h	Antiangiogenic	[100]
*Crotalus* oreganus oreganus (rattlesnake)	Myotoxin‐3 (45)	U87	1–50 µM	24 h	Antimicrotubule	[101]
*Micrurus lemniscatus* (snake)	β‐micrustoxin (NR)	U138 and U251	0.2, 2, 20, 200 nM	3, 12 and 24 h	Apoptosis ↑ p53, p21 and p27	[102]
*Vipera* lebetina obtuse (snake)	VLO4 (66)	LN18 and LN229	0.05 and 0.1 µM	24 h	Antiangiogenic	[103]

*Note:* This table presents the in vitro evaluation of snake venom–derived peptides with activity against glioblastoma. Listed data include the source species, peptide name and amino acid length, glioblastoma cell lines tested, applied dose, treatment duration, and the observed biological responses. Data were compiled from the cited references.

Abbreviations: aa, amino acids; NR, not reported.

The envenomation caused by *Ma. lebetina trasmediterranea* is characterized by severe clinical complications such as edema, hemorrhage, local tissue damage, hemolysis, muscle necrosis, and abnormalities in the blood coagulation system. The mixture of proteins and peptides present in the venom is responsible for these complications [104]. Among the peptides isolated from this venom, PIVL (67‐mer) has been shown to inhibit the adhesion, migration, and invasion of glioblastoma cells. Its antitumor effect has been associated with interference with integrin receptor function, which specifies the Kunitz/BPTI inhibitor family [99].

The venom of *A. contortrix* has also been studied. This snake is found in the forests of the southeastern part of the United States and is considered one of the most common venomous snakes in that country [105]. A peptide isolated from its venom, named contortrostatin (65‐mer), inhibited the adhesion and invasion of the glioblastoma cell line by interacting with several integrins, including αvβ 3, αβ5, and α5β1 [97]. In another study carried out by Pyrko et al. (2005), contortrostatin exerted cytotoxic activity and was safe to administer by intracranial injection, as it did not induce cerebral hemorrhage or provoke an immune response in the brains of rodents [98]. Another peptide isolated from the same snake venom, vicrostatin (69‐amino acid), sensitized glioblastoma cells to chemotherapy, indicating its potential use in combination therapies [100]. The Myotoxin‐3 peptide (45‐mer), isolated from the venom of the rattlesnake *C. oreganus oreganus*, exerted cytotoxic activity, in addition to affecting the dynamics of microtubules and inducing cells to lose their elongated morphology to a rounded morphology in glioblastoma cells (U87) [101].

Another mechanism observed for peptides originating from snake venom is one of the β‐microtoxins, a svPLA_2_ from *M. lemniscatus* venom, which acts over tumor suppressor p53 and two cyclin‐dependent kinase inhibitors, p21 and p27 [102]. VLO4 peptide (66‐amino acid) inhibited angiogenesis by interacting with the α5β1 integrin, which is expressed in all the glioma cell lines tested [103].

**Figure 4:**
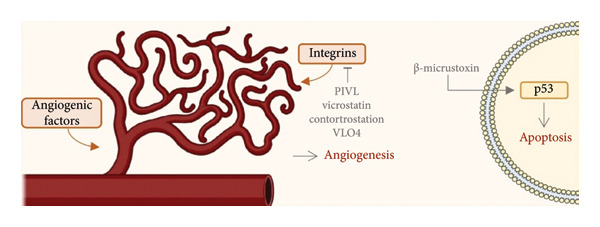
Venom‐derived peptides from snake modulate molecular events in glioblastoma cells in vitro. Abbreviations: ↓, upregulation; T inhibit; p53: tumor protein p53. Image provided by Servier Medical Art (https://smart.servier.com/), licensed under CC BY 4.0 (https://creativecommons.org/licenses/by/4.0/).

In summary, the majority of snake‐derived peptides exhibit antiangiogenic activity (Figure 4), as observed with contortrostatin, PIVL, vicrostatin, and VLO4, suggesting a shared mechanism targeting tumor vascularization and integrin signaling. Myotoxin‐3 showed an antimicrotubule effect on U87 cells, whereas β‐micrustoxin induced apoptosis in U138 and U251 cells through the upregulation of p53, p21, and p27, highlighting the involvement of cell‐cycle arrest and mitochondrial signaling. Overall, these diverse actions underscore the multifunctional nature of snake venom peptides, encompassing antiangiogenic, apoptotic, and cytoskeletal‐disrupting mechanisms. Their longer length and disulfide‐rich architectures confer structural stability and receptor specificity, making them promising scaffolds for antiglioblastoma drug development.

### 3.4. An In Silico Perspective on the Anticancer Potential and Toxicity of Venom‐Derived Peptides With Antiglioblastoma Properties

While in vitro and in vivo studies have clearly demonstrated the antiglioblastoma potential of the peptides presented here, significant barriers remain in their preclinical and clinical development, in crucial aspects such as selectivity, toxicity, and therapeutic efficacy. Under this preamble, in silico approaches emerge as key tools to complement and enhance this research, in order to advance these therapeutic candidates to new stages of evaluation. The computational prediction of critical properties such as anticancer activity, systemic toxicity, or hemolysis not only allows for a more in‐depth characterization of these molecules but also enables the selection and prioritization of candidates with a greater likelihood of clinical success. This predictive approach constitutes the central axis of this work, integrating bioinformatics analysis and AI tools (machine/deep learning models and algorithms) to revalue and expand existing knowledge from a more strategic and forward‐looking perspective.

In recent years, bioinformatics tools and AI have gained significant relevance in the ongoing search for new therapeutic alternatives for various diseases [106, 107]. Therefore, they have become essential resources for pharmaceutical and biotechnological research [108]. The power of these tools lies in their ability to predict (with increasing efficiency) physical, chemical, and structural properties; potential cellular targets; and mechanisms of action, ligands, and even effective concentrations [109–111].

All of this has been achieved both through the constant updating of numerous databases (through the discovery of new compounds) and the development of increasingly sophisticated algorithms, mainly supervised by machine learning models such as support vector machines (SVMs), random forest, and deep neural networks [112, 113]. In the field of peptides, this development has been particularly significant, as reflected in the various tools available to predict toxicity [36], hemolysis [34, 114], and antitumor activity [115], among other properties or cellular targets [116]. This allows for the identification of promising candidates with a greater likelihood of success in the different in vitro and in vivo experimental phases, which also translates into optimized resources and time. To validate the potential of the animal venom–derived peptides against different types of glioma presented here, and to gain insights beyond the results already generated, predictions of their toxicity, hemolytic and BPP properties, and anticancer potential were made using freely available bioinformatics and AI tools. These results are presented in Tables 6 and 7 and discussed below.

**Table 6:** Multilayered analysis of the activity and toxicity of venom‐derived peptides leveraging computational methods.

Animal group	Peptide name	HemoPi2		HLPpred‐fuse		Toxin pred 3.0	CSM toxicity		pkCMS		AntiCP 2.0		MLACP 2.0	
		HC_50_ (μM)	Category	First prediction (value)	Second prediction (value)	Category (score)	Prediction	Hydrophobicity	AMES toxicity	Hepatoxicity	Score	Prediction	Probability	Class
Arachnids	Psalmotoxin 1	46.32	HLP	HLP (0.56)	Low (0.23)	Toxin (1.0)	Nontoxin	−0.91	No	No	0.58	ACP	0.316	Non‐ACP
	Phα1β	47.04	HLP	Non‐HLP (0.27)	—	Nontoxin (0.34)	Nontoxin	−0.48	No	No	0.58	ACP	(‐)	(‐)
	LyeTxI‐b	73.62	HLP	HLP (0.99)	High (0.89)	Toxin (0.895)	Nontoxin	0.29	No	Yes	0.45	ACP	0.929	ACP
	Chlorotoxin	43.19	HLP	Non‐HLP (0.14)	—	Toxin (1.0)	Nontoxin	−0.55	No	Yes	0.58	ACP	0.495	Non‐ACP
	BmKCT	21.96	HLP	HLP (0.67)	High (0.56)	Nontoxin (0.215)	Toxin	0.52	No	No	0.58	ACP	(‐)	(‐)
	AaCTx	45.59	HLP	HLP (0.65)	Low (0.24)	Toxin (1.0)	Nontoxin	0.02	No	Yes	0.2	Non‐ACP	0.782	ACP
	Acra3	38.70	HLP	Non‐HLP (0.45)	—	Toxin (0.675)	Nontoxin	−0.13	No	No	0.58	ACP	(‐)	(‐)
	KAaH1	47.10	HLP	Non‐HLP (0.27)	—	Toxin (0.6)	Nontoxin	−0.36	No	No	0.58	ACP	(‐)	(‐)
	KAaH2	44.87	HLP	Non‐HLP (0.04)	—	Toxin (1.0)	Nontoxin	−0.42	No	No	0.58	ACP	(‐)	(‐)
	RK	59.87	HLP	Non‐HLP (0.00)	—	Toxin (1.0)	Nontoxin	−0.06	No	Yes	0.44	Non‐ACP	0.146	Non‐ACP
	RK1	127.17	Non‐HLP	Non‐HLP (0.01)	—	Toxin (1.0)	Nontoxin	0.13	No	Yes	0.53	ACP	0.094	Non‐ACP
	AaTs‐1	186.89	Non‐HLP	(‐)	(‐)	Toxin (0.89)	Nontoxin	−0.45	No	Yes	0.63	ACP	0.929	ACP
	P01	106.18	Non‐HLP	Non‐HLP (0.01)	—	Toxin (0.92)	Nontoxin	−0.7	No	Yes	0.33	Non‐ACP	0.069	Non‐ACP
	Androcin 18−1	16.21	HLP	HLP (0.95)	High (0.74)	Nontoxin (0.2)	Nontoxin	0.3	No	Yes	0.48	ACP	0.848	ACP

Insects	Anoplin	508.26	Non‐HLP	HLP (1.00)	Low (0.37)	Nontoxin (0.065)	Nontoxin	0.63	No	Yes	0.0	Non‐ACP	0.937	ACP
	MP1	2.96	HLP	HLP (1.00)	High (0.86)	Nontoxin (0.0)	Nontoxin	0.06	No	Yes	0.48	ACP	0.897	ACP
	MPX	107.19	Non‐HLP	HLP (1.00)	High (0.99)	Toxin (0.49)	Toxin	0.53	No	Yes	0.0	Non‐ACP	0.89	ACP
	HR1	117.05	Non‐HLP	HLP (1.00)	High (0.60)	Nontoxin (0.32)	Nontoxin	1.36	No	Yes	0.28	Non‐ACP	0.897	ACP
	Melittin	9.92	HLP	HLP (0.66)	Low (0.48)	Toxin (0.5)	Nontoxin	0.31	No	Yes	0.48	ACP	0.842	ACP
	Apamin	144.022	Non‐HLP	Non‐HLP (0.13)	—	Toxin (0.755)	Nontoxin	−0.73	No	No	0.31	Non‐ACP	0.503	ACP

Snakes	Contortrostatin	64.61	HLP	HLP (0.63)	Low (0.24)	Nontoxin (0.355)	Nontoxin	−0.49	No	No	0.58	ACP	(‐)	(‐)
	Vicrostatin	61.64	HLP	Non‐HLP (0.46)	—	Nontoxin (0.335)	Nontoxin	−0.57	No	No	0.58	ACP	(‐)	(‐)
	Myotoxin‐3	46.96	HLP	HLP (0.91)	Low (0.31)	Toxin (0.825)	Nontoxin	−0.8	No	Yes	0.58	ACP	(‐)	(‐)
	VLO4	55.98	HLP	HLP (1.00)	High (0.67)	Nontoxin (0.32)	Nontoxin	−0.81	No	Yes	0.58	ACP	(‐)	(‐)

*Note:* Several tools were used for an integral view of the selectivity of venom‐derived peptides. The table shows the results of HemoPi2 and HLPpred‐Fuse tools for hemolytic activity; ToxinPred 3.0, CSM toxicity, and pkCSM pharmacokinetics for toxic activity; and AntiCP 2.0 and MLACP 2.0 for anticancer activity. The colors represent the different activities of the animal venom–derived peptides predicted by the different computational tools: Red indicates the hemolytic activity, blue represents the nonhemolytic activity, and pink is for those peptides that were recognized as hemolytic for the first prediction of HLPpred‐Fuse, but low hemolytic by the second one. Gray denotes the toxic activity, orange represents the nontoxic activity, yellow is for the nonanticancer activity, and green signifies the anticancer activity. HLP, hemolytic peptide; Non‐HLP, nonhemolytic peptide; HC50, minimum hemolytic concentration; ACP, anticancer peptide; Non‐ACP, non‐anticancer peptide, (–): not performed, —: no result.

**Table 7:** *In silico* prediction of blood–brain barrier penetrating properties of venom‐derived peptides.

Animal group	Peptide	BBBper	LogBB_Pred	
			logBB	BBB
Arachnids	Psalmotoxin 1	Nonpermeable	−1.24677	Nonpermeable
	Phα1β	—	−1.18235	Nonpermeable
	LyeTxI‐b	—	−0.86123	Permeable
	Chlorotoxin	Nonpermeable	−1.16743	Nonpermeable
	BmKCT	—	−1.00077	Nonpermeable
	AaCTx	—	−1.06288	Nonpermeable
	Acra3	—	−1.21264	Nonpermeable
	KAaH1	—	−1.26405	Nonpermeable
	KAaH2	—	−1.26824	Nonpermeable
	RK	—	−1.10991	Nonpermeable
	RK1	—	−0.89971	Permeable
	AaTs‐1	—	−1.09001	Nonpermeable
	P01	—	−1.1815	Nonpermeable
	Androcin 18−1	—	−0.95476	Permeable

Insects	Anoplin	Nonpermeable	−0.67328	Permeable
	MP1	Nonpermeable	−1.02185	Nonpermeable
	MPX	Nonpermeable	−0.93172	Permeable
	HR1	Nonpermeable	−0.65683	Permeable
	Melittin	—	−1.02457	Nonpermeable
	Apamin	Nonpermeable	−1.14236	Nonpermeable

Snakes	Contortrostatin	—	−1.18069	Nonpermeable
	Vicrostatin	—	−1.17823	Nonpermeable
	Myotoxin‐3	—	−1.24558	Nonpermeable
	VLO4	—	−1.15548	Nonpermeable

*Note:* Computational tools were used to classify peptides according to their predicted BBB penetration potential. Purple corresponds to nonpermeable peptides, while turquoise indicates the BBB‐permeable peptides. —: It was not possible to perform, due to the limitations of the tool; BBB permeable: LogBB ≥ −1.

Bioinformatic tools serve as indispensable resources for identifying key characteristics and properties of molecules, empowering researchers to thoroughly evaluate their potential as therapeutic candidates [107]. Over the years, these tools have undergone significant refinement, resulting in more robust and reliable predictive capabilities. A major advantage of bioinformatics lies in its ability to optimize time and resources by enhancing the precision of technical efforts and streamlining testing and validation processes [106, 107]. However, it is essential to acknowledge that these tools, while powerful, are not definitive but rather complementary to laboratory experiments. As shown by the outcomes of various tools applied for the same purpose, discrepancies may occur, leading to varying results [117, 118]. Such differences require careful analysis and interpretation to ensure accurate conclusions and a holistic understanding.

Although many peptides can be highly effective in inhibiting tumor cells in vitro, it is also very important to perform assays that determine the activity of these molecules on nontumor cells [119, 120]. This is done to determine the selectivity of these molecules and, consequently, strengthen their role as promising therapeutic candidates. Unfortunately, this critical aspect is often overlooked in many studies investigating newly identified molecules with pharmacological potential. Consequently, the rapid development and expanding capabilities of computational tools make them invaluable resources for the prediction and characterization of a wide range of biological activities.

Cytotoxicity assays are essential steps in the characterization of novel drug candidates that will come into contact with biological systems at the in vivo scale [121]. In the field of peptide‐drug development, this evaluation is of great importance, and there are numerous options for achieving it [122]. However, hemolysis assays stand out for their advantages, such as their low cost, accessibility, and ease of implementation [123]. If the compound under study causes hemolysis, hemoglobin and other cellular components will be released into the supernatant, and due to the hemoglobin absorbance spectrum, the degree of hemolysis can be determined by spectrophotometric methods [124]. Although there may be significant differences between the different hemolysis protocols, such as the organism of origin of the blood used, sample treatment, selection of appropriate positive controls, or even the analysis methods, the aforementioned advantages that distinguish this technique remain [125, 126]. In the field of peptides, the practicality of the hemolysis assay has expanded its scope to include the evaluation of large quantities of peptides. This, in turn, has led to the creation of various databases and highly efficient prediction tools for hemolytic activity. The HemoPi2 and HLPpred‐Fuse tools were used in this work.

HemoPi2 uses machine learning based on the physicochemical and structural properties of peptides and has a robust dataset of 1926 experimentally validated hemolytic peptides [34]. On the other hand, HLPpred‐Fuse employs a deep learning approach that integrates multiple sequence features, such as amino acid index (AAI), binary profile (BPF), composition–transition–distribution (CTD), conjoint triad (CTF), quasisequence order (QSO), and grouped dipeptide composition (GDPC), among others [35]. These differences in methodology mean that Hemopi2 generates an IC_50_ value for the peptide, in addition to its respective category (HLP or non‐HLP), and HLPpred‐Fuse, working with a two‐layer framework model, generates two predictions at the same time. The latter refers to the fact that if the peptide is recognized as HLP in the first framework, the second will determine how high this activity is likely to be.

In this case, of the 26 peptides derived from animal venoms here presented, 11 positive matches were found for hemolysis (peptides recognized as hemolytic by both tools) and three negative matches (nonhemolytic). Eleven discrepancies were found. The AaTs‐1 peptide could only be evaluated by HemoPi2. Of the 11 positive matches found, it can be observed that the HC_50_ concentration range (generated by HemoPi2) is 2.96–73.62 μM. The three negative matches showed HC_50_ values > 100 μM. It is best to highlight those peptides that were recognized as non‐HLP by both tools. These were RK1, P01, both derived from arachnid venoms, and apamin, derived from bee venom. Also, anoplin peptides (insect) can be added, which were cataloged as non‐HLP by Hemopi2 (HC_50_ = 508.26 μM), and despite being recognized as HLP by the first HLPpred‐Fuse framework, the second indicated that this probable activity was low. All other peptides were recognized as hemolytic, either by one tool or by both. This represents a major limitation, as highly hemolytic peptides have low selectivity, exhibit systemic toxicity, and are poorly delivered [127].

ToxinPred 3.0 and CSM toxicity both classify peptides as either toxic or nontoxic; however, they differ in their approaches. ToxinPred 3.0 provides a quantitative toxicity score, where values greater than 0.45 indicate toxicity for all peptides identified as toxic. Interestingly, only the MPX peptide was consistently recognized as toxic by both tools. In contrast, eight peptides were predicted to be nontoxic by both models, while 17 peptides showed conflicting classifications. This discrepancy highlights the divergence between the models, likely arising from differences in their training datasets, underlying algorithms, feature extraction methods, and threshold ranges for toxicity determination. ToxinPred 3.0 utilizes a model that integrates structural and sequence‐based features derived from experimentally characterized toxic peptides [36]. When combining hemolytic and toxicity predictions, anoplin emerges as a promising candidate, classified as nontoxic and exhibiting low hemolytic potential.

On the other hand, CSM toxicity and pkCSM use quantitative structure–activity relationship (QSAR) modeling approaches and machine learning trained on extensive chemical and pharmacokinetic databases. These tools analyze the three‐dimensional structure of compounds to provide information on charge distribution, surface areas, bonds, and other properties. The QSAR model is trained on large databases of experimentally validated toxic compounds, allowing for pattern recognition. In addition, CSM toxin displays the number of amino acids of each type within the sequence (aliphatic, aromatic, polar) as well as properties such as hydrophobicity and molecular weight [37]. Most peptides derived from animal venoms (24 peptides) were recognized as nontoxic by this tool. Of the eight negative matches between these tools, there is no good consensus with the hemolysis results either. Phα1, anoplin, HR1, and vicrostatin peptides stand out, since they were recognized as nontoxic by both tools and hemolytic by only one of the predictors used for this activity.

In addition, hydrophobicity values generated by CSM toxicity were also included in the table. Peptides with high hydrophobicity can penetrate the core membrane of cancer cells, causing their destruction and cell death via necrosis [128]. A close relationship has been shown between the hydrophobic property of a peptide and its anticancer potential. An example can be seen in the study carried out by Huan et al. (2011) [129], in which the increase in hydrophobicity also increases the anticancer activity and the selectivity of cationic peptides. However, there is always a threshold in the increase of hydrophobic properties, which, if exceeded, results in toxicity toward normal cells [130].

Finally, the pkCSM tool is used to predict the pharmacokinetic and toxicokinetic properties of different types of molecules. Since this is a tool that determines specific toxicity parameters, it is not included in the comparison with other predictors. To work with this tool, the structures of the molecules must be placed in SMILES format. This bioinformatics resource is focused on evaluating key aspects of absorption, distribution, metabolism, excretion, and toxicity (ADME‐Tox) [38]. In this case, it focused on two parameters: AMES toxicity and hepatoxicity. AMES toxicity refers to the mutagenic capacity of a compound and therefore its capacity to act as a carcinogen. In this case, all the peptides analyzed do not have AMES toxicity; therefore, they are not carcinogenic. Hepatoxicity is a key aspect in the discovery of a new drug and is usually decisive in the continuation in the development stages. The pkCSM model for this purpose was built on the basis of data from 531 compounds that have been shown to cause liver side effects in humans; 15 of the 26 animal venom peptides (61%) were categorized as positive for hepatotoxicity.

Finally, predictions of the peptides’ antitumor activity were made using AntiCP 2.0 and MLACP 2.0. Both generate a classification (ACP or non‐ACP), and a score and probability value, respectively. A limitation of MLACP 2.0 is that it cannot perform predictions for peptides longer than 50 amino acid residues. With this in mind, five positive matches (ACP), two negative matches (non‐ACP), and nine discrepancies were obtained. Of the matches found, only the AaTs‐1 peptide stands out, as in addition to being recognized by ACP using both tools, it was also classified as nonhemolytic (HemoPi 2.0) and nontoxic (CSM toxicity). However, pkCMS classified it as hepatoxic. MLACP 2.0 was developed using a training dataset and a wide range of encodings and models using different models. Each model was ranked based on its performance and subsequently trained using a convolutional neural network (CNN). The result of this process was the predictor, which has shown excellent performance during cross‐validation and independent evaluation compared to CNN‐based embedding models and conventional standalone models [40]. On the other hand, AntiCP 2.0 is a model developed with a main dataset of 861 experimentally validated anticancer peptides and 861 non‐anticancer or validated antimicrobial peptides. The alternative dataset comprises 970 anticancer peptides and 970 non‐anticancer peptides (randomly selected from Swiss‐Prot). This predictor uses machine learning models, specifically SVM algorithms, to predict peptides with anticancer activity [39].

The results in Table 6 demonstrate the potential of different bioinformatics and AI tools to predict various activities and functions of antiglioblastoma peptides. However, they also reveal inherent limitations in the use of these in silico resources. The discrepancies between different tools are notable. While some models classify certain peptides as toxic or hemolytic, others consider them safe. This reflects the differences in the training sets, algorithms, and data types employed by each tool. This variability requires that results be considered indicative, not definitive, and that they always be integrated with experimental validations. Furthermore, differences in hydrophobicity also suggest a relevant role for this property between efficacy and toxicity, as has been discussed by several studies [131–133].

In our comparative analysis, we also observed discrepancies between experimental data and in silico predictions across several venom‐derived peptides. These inconsistencies likely stem from the limitations of current prediction algorithms, which are built on machine learning models trained with biased or incomplete datasets. Most tools infer activity primarily from physicochemical features, such as charge, hydrophobicity, and amino acid composition, rather than from mechanistic or structural determinants. Consequently, they may tend to overestimate activity for highly cationic or amphipathic sequences and underestimate peptides whose effects depend on receptor binding, channel modulation, or complex secondary structures. For instance, PhTx3‐3 was experimentally shown to be active in vivo but was predicted as hemolytic and non‐anticancer, possibly because its ion‐channel modulation mechanism is not captured by sequence‐based classifiers. Conversely, anoplin was predicted to be inactive despite in vitro anticancer effects, reflecting its context‐dependent membrane interactions. Similarly, melittin, known for its strong hemolytic and cytolytic activity, was inaccurately classified as low hemolytic, likely due to dataset‐driven bias toward short or mildly amphipathic peptides. Finally, Apamin, a disulfide‐rich neurotoxin with documented anticancer properties, was predicted as non‐anticancer, illustrating how compact, structured peptides with receptor‐specific actions fall outside the predictive scope of current models. Collectively, these findings underscore that AI‐based prediction tools, while informative, cannot yet fully capture the multimechanistic nature of venom‐derived peptides and should therefore be interpreted as supportive rather than definitive evidence when evaluating therapeutic potential.

#### 3.4.1. Computational Prediction of BBB Permeability of Antiglioblastoma Peptides

Effective drug delivery across the natural protective membrane (BBB) remains one of the greatest challenges in the development of antiglioblastoma therapeutics [134]. The BBB’s tightly regulated endothelial structure restricts the entry of most biomolecules, including large or highly polar compounds, thereby limiting the efficacy of many potential anticancer agents [135]. Understanding the BBB permeability properties of candidate peptides is therefore crucial for assessing their potential as therapeutics for glioblastoma, a malignancy located within the central nervous system [136]. Peptides capable of penetrating the BBB can directly reach tumor sites, whereas nonpermeable molecules may require delivery systems or conjugation strategies to achieve therapeutic concentrations in the brain [137].

In this review, the BBB permeability of animal venom–derived antiglioblastoma peptides was evaluated using two complementary computational tools: BBBper and LogBB_Pred. Both predictors estimate the ability of peptides to cross the BBB based on physicochemical and structural descriptors such as hydrophobicity, charge, and molecular size. BBBper provides categorical outputs (permeable or nonpermeable), while LogBB_Pred calculates the logarithmic blood–brain concentration ratio (*logBB*), with values higher than −1.0 generally indicating potential permeability. The combination of both tools allowed for a valuable assessment of the peptides’ theoretical capacity to access the central nervous system.

Comparison between the two predictive models revealed a generally consistent trend, with the majority of peptides classified as nonpermeable across both tools. Among arachnid‐derived peptides, most displayed *logBB* values below −1.0, indicating low likelihood of BBB penetration. Only LyeTxI‐b, RK1, and Androcin 18−1 were predicted to be BBB permeable, suggesting that their smaller size and amphipathic characteristics may facilitate passive translocation. For insect‐derived peptides, the predictions were more variable. BBBper categorized most peptides (MP1, MPX, HR1, Apamin) as nonpermeable, while LogBB_Pred identified anoplin, MPX, and HR1 as potentially permeable, with *logBB* values between −0.6 and −0.9. This partial discrepancy likely reflects structural borderline cases, where moderate hydrophobicity and shorter chain length may influence passive diffusion potential. In contrast, melittin was consistently predicted as nonpermeable by both models. All snake‐derived peptides, including contortrostatin, vicrostatin, Myotoxin‐3, and VLO4, were uniformly predicted to be nonpermeable by both predictors, with *logBB* values below −1.15, indicating a low probability of passive diffusion across the BBB.

Collectively, the in silico predictions indicate that only 6 out of 23 peptides (≈26%) were classified as BBB‐permeable by at least one of the computational models, while 17 peptides (≈74%) were considered nonpermeable. The LogBB_Pred tool yielded slightly more permissive predictions than BBBper, although both showed a high degree of overall agreement. These findings suggest that most venom‐derived antiglioblastoma peptides exhibit limited intrinsic BBB permeability. However, a subset particularly short, amphipathic peptides from arachnid and insect origins, may possess favorable physicochemical traits that enable partial BBB penetration. These results highlight the need for rational design and delivery optimization, such as conjugation with transport ligands or nanoparticle encapsulation, to improve brain bioavailability in future antiglioblastoma peptide therapeutics. On the other hand, this also underscores the importance of in vivo validation, as some AI‐based predictive models may be biased or fail to accurately capture the BBB permeability and other pharmacokinetic properties of venom‐derived peptides.

## 4. Future Perspectives on the Use of Venom‐Derived Peptides in Glioblastoma

A major obstacle in the development of antiglioblastoma drugs is their limited ability to cross the BBB [136]. Our computational findings confirm this trend. The results indicate that most peptides exhibit low predicted BBB permeability, which could significantly restrict their therapeutic potential for brain tumors. Nevertheless, these predictions require experimental validation, as in silico tools may not fully capture the complexity of in vivo transport mechanisms. Future research should therefore prioritize in vivo studies and more physiologically relevant models that go beyond traditional monolayer cell assays, to better evaluate the pharmacokinetic and pharmacodynamic behavior of these venom‐derived peptides within the central nervous system. Inspiration can be drawn from CPPs that have already progressed to Phase I and II clinical trials, which demonstrate how structural optimization and delivery strategies can improve BBB penetration and guide the translational development of antiglioblastoma peptides [29].

AI is rapidly transforming peptide discovery and optimization, offering valuable tools for predicting bioactivity, toxicity, and BBB permeability [111]. In this study, we identified several AI‐based prediction platforms with promising utility; however, discrepancies between tools and especially divergences between computational predictions and experimental findings highlight the current limitations of these algorithms. One of the major constraints is the limited size of venom‐derived peptide datasets, which hampers model training and reduces predictive reliability. To overcome these challenges, future efforts should focus on high‐throughput screening and systematic data collection, expanding the libraries of venom‐derived antiglioblastoma peptides. Such enriched datasets would enable the development of more accurate AI models capable of guiding rational design and improving translational predictability.

Our analysis also revealed a limited degree of chemical modification and engineering among venom‐derived peptides tested against glioblastoma, as well as a lack of evaluation in realistic preclinical models. Since native (L‐amino acid) peptides are often susceptible to proteolytic degradation and have short half‐lives, structural optimization is essential to enhance their therapeutic viability. Lessons from successful peptide‐based drugs already in clinical use in different fields, including oncology, demonstrate that chemical modifications, such as cyclization, PEGylation, or incorporation of non‐natural amino acids, can significantly improve stability, bioavailability, and target specificity [44]. Integrating such engineering strategies into the discovery and development workflow of venom‐derived peptides will be critical to achieving effective and translatable antiglioblastoma therapies in the future.

## 5. Conclusions

This work presents a comprehensive synthesis of current knowledge on animal venom–derived peptides with therapeutic potential against glioblastoma, one of the most aggressive and treatment‐resistant brain cancers. By integrating data from in vitro and in vivo studies, we identified key peptides from spiders, scorpions, insects, and snakes, detailing their effective concentrations, exposure times, and proposed mechanisms of action. This compilation not only offers a panoramic view of the state of research in this field but also establishes a reference framework for selecting and prioritizing promising candidates for further pharmacological development.

A major contribution of this study lies in the integration of bioinformatics and AI‐based predictive tools to complement experimental evidence (in vitro and in vivo findings). While certain discrepancies between prediction tools were observed, the comparative approach enhanced our understanding of key structural and functional trends. Additionally, divergences between experimental findings and computational outcomes were identified, suggesting room for methodological optimization and model improvement.

Looking forward, the convergence of AI‐driven molecular design, automated peptide synthesis, and high‐precision experimental validation will be instrumental in advancing venom‐derived peptides toward antiglioblastoma therapies. Collectively, these strategies pave the way for the rational design of safe, effective, and target‐specific peptide therapeutics, marking a crucial step toward innovative treatments for glioblastoma patients.

## Ethics Statement

The authors have nothing to report.

## Conflicts of Interest

The authors declare no conflicts of interest.

## Author Contributions

All authors contributed to the conception and design of the work.

## Funding

The present study was supported by the UFSJ, CAPES, FAPEMIG, and CNPq for their financial support.

## References

[1] Xu S. , Zhang G. , Zhang J. , Liu W. , Wang Y. , and Fu X. , Advances in Brain Tumor Therapy Based on the Magnetic Nanoparticles, International Journal of Nanomedicine. (2023) 18, 7803–7823, 10.2147/ijn.s444319. 38144513 PMC10749175

[2] Xu S. , Tang L. , Li X. , Fan F. , and Liu Z. , Immunotherapy for Glioma: Current Management and Future Application, Cancer Letters. (2020) 476, 1–12, 10.1016/j.canlet.2020.02.002. 32044356

[3] Yang K. , Wu Z. , Zhang H. et al., Glioma Targeted Therapy: Insight Into Future of Molecular Approaches, Molecular Cancer. (2022) 21, no. 1, 10.1186/s12943-022-01513-z. PMC8822752 35135556

[4] Yang Y. , Wang J. , Shi F. , Shan A. , Xu S. , and Lv W. , BDKRB2 is a Novel EMT-Related Biomarker and Predicts Poor Survival in Glioma, Aging (Albany NY). (2021) 13, no. 5, 7499–7516, 10.18632/aging.202614. 33686021 PMC7993731

[5] Weller M. , Wen P. Y. , Chang S. M. et al., Glioma, Nature Reviews Disease Primers. (2024) 10, no. 1, 10.1038/s41572-024-00516-y. 38724526

[6] Silvani A. , New Perspectives: Glioma in Adult Patients, Tumori. (2023) 109, no. 4, 350–355, 10.1177/03008916231159716. 36964665

[7] Angom R. S. , Nakka N. M. R. , and Bhattacharya S. , Advances in Glioblastoma Therapy: An Update on Current Approaches, Brain Sciences. (2023) 13, no. 11, 10.3390/brainsci13111536. PMC10669378 38002496

[8] Khan M. , Nasim M. , Feizy M. et al., Contemporary Strategies in Glioblastoma Therapy: Recent Developments and Innovations, Neuroscience. (2024) 560, 211–237, 10.1016/j.neuroscience.2024.09.022. 39368608

[9] Conte L. , Caruso G. , Philip A. K. et al., Artificial Intelligence-Assisted Drug and Biomarker Discovery for Glioblastoma: A Scoping Review of the Literature, Cancers (Basel). (2025) 17, no. 4, 10.3390/cancers17040571. PMC11852502 40002166

[10] Rončević A. , Koruga N. , Soldo Koruga A. , and Rončević R. , Artificial Intelligence in Glioblastoma-Transforming Diagnosis and Treatment, Chinese Neurosurgical Journal. (2025) 11, no. 1, 10.1186/s41016-025-00399-2. PMC12128298 40457483

[11] Almeida J. R. , Pinos-Tamayo E. A. , Mendes B. et al., Snake Venom-Derived Peptides as Anticancer Candidates: Pioneering Next-Generation Therapies, Biochimica et Biophysica Acta (BBA)-Reviews on Cancer. (2025) 1880, no. 6, 10.1016/j.bbcan.2025.189479. 41110776

[12] Mohamed Abd El-Aziz T. , Garcia Soares A. , and Stockand J. D. , Snake Venoms in Drug Discovery: Valuable Therapeutic Tools for Life Saving, Toxins. (2019) 11, no. 10, 10.3390/toxins11100564, 2-s2.0-85072695699. PMC6832721 31557973

[13] Hilchie A. L. , Hoskin D. W. , and Power Coombs M. R. , Anticancer Activities of Natural and Synthetic Peptides, Advances in Experimental Medicine and Biology. (2019) 1117, 131–147, 10.1007/978-981-13-3588-4_9, 2-s2.0-85064664349. 30980357

[14] Simoes-Silva R. , Alfonso J. , Gomez A. et al., A Natural Library of New Potential Therapeutic Molecules: Challenges and Current Perspectives, Current Pharmaceutical Biotechnology. (2018) 19, no. 4, 308–335, 10.2174/1389201019666180620111025, 2-s2.0-85053808489. 29929461

[15] de Oliveira A. N. , Soares A. M. , and da Silva S. L. , Peptides From Animal Venom and Poisons, International Journal of Peptide Research and Therapeutics. (2023) 29, no. 5, 10.1007/s10989-023-10557-8.

[16] Orozco-Mera J. , Montoya-Gómez A. , Lopes D. S. , and Jiménez-Charris E. , Snake Venom Bioprospecting as an Approach to Finding Potential Anti-Glioblastoma Molecules, Journal of Venomous Animals and Toxins including Tropical Diseases. (2024) 30, 10.1590/1678-9199-jvatitd-2024-0015. PMC11404105 39285908

[17] Majc B. , Novak M. , Lah T. T. , and Križaj I. , Bioactive Peptides From Venoms Against Glioma Progression, Frontiers in Oncology. (2022) 12, 10.3389/fonc.2022.965882. PMC9476555 36119523

[18] Almeida J. R. , Resende L. M. , Watanabe R. K. et al., Snake Venom Peptides and Low Mass Proteins: Molecular Tools and Therapeutic Agents, Current Medicinal Chemistry. (2017) 24, no. 30, 3254–3282, 10.2174/0929867323666161028155611, 2-s2.0-85037847566. 27804880

[19] Pennington M. W. , Czerwinski A. , and Norton R. S. , Peptide Therapeutics From Venom: Current Status and Potential, Bioorganic & Medicinal Chemistry. (2018) 26, no. 10, 2738–2758, 10.1016/j.bmc.2017.09.029, 2-s2.0-85030644516. 28988749

[20] Coulter-Parkhill A. , McClean S. , Gault V. A. , and Irwin N. , Therapeutic Potential of Peptides Derived From Animal Venoms: Current Views and Emerging Drugs for Diabetes, Clinical Medicine Insights: Endocrinology and Diabetes. (2021) 14, 10.1177/11795514211006071. PMC8491154 34621137

[21] Marchi F. C. , Mendes-Silva E. , Rodrigues-Ribeiro L. , Bolais-Ramos L. G. , and Verano-Braga T. , Toxinology in the Proteomics Era: A Review on Arachnid Venom Proteomics, Journal of Venomous Animals and Toxins including Tropical Diseases. (2022) 28, 10.1590/1678-9199-jvatitd-2021-0034. PMC8893269 35291269

[22] Himaya S. W. A. and Lewis R. J. , Venomics-Accelerated Cone Snail Venom Peptide Discovery, International Journal of Molecular Sciences. (2018) 19, no. 3, 10.3390/ijms19030788, 2-s2.0-85043597619. PMC5877649 29522462

[23] Guan C. , Torres M. D. T. , Li S. , and de la Fuente-Nunez C. , Computational Exploration of Global Venoms for Antimicrobial Discovery With Venomics Artificial Intelligence, Nature Communications. (2025) 16, no. 1, 10.1038/s41467-025-60051-6. PMC12254355 40645962

[24] Almeida J. R. , Mendes B. , Lancellotti M. et al., Lessons From a Single Amino Acid Substitution: Anticancer and Antibacterial Properties of Two Phospholipase A2-Derived Peptides, Current Issues in Molecular Biology. (2022) 44, no. 1, 46–62, 10.3390/cimb44010004. PMC8929095 35723383

[25] Robles-Loaiza A. A. , Pinos-Tamayo E. A. , Mendes B. et al., Peptides to Tackle Leishmaniasis: Current Status and Future Directions, International Journal of Molecular Sciences. (2021) 22, no. 9, 10.3390/ijms22094400. PMC8122823 33922379

[26] Al Musaimi O. , AlShaer D. , de la Torre B. G. , and Albericio F. , 2024 FDA TIDES (Peptides and Oligonucleotides) Harvest, Pharmaceuticals. (2025) 18, no. 3, 10.3390/ph18030291. PMC11945313 40143070

[27] Al Musaimi O. , FDA’s Stamp of Approval: Unveiling Peptide Breakthroughs in Cardiovascular Diseases, ACE, HIV, CNS, and Beyond, Journal of Peptide Science. (2024) 30, no. 11, 10.1002/psc.3627. 38885943

[28] Marqus S. , Pirogova E. , and Piva T. J. , Evaluation of the Use of Therapeutic Peptides for Cancer Treatment, Journal of Biomedical Science. (2017) 24, no. 1, 10.1186/s12929-017-0328-x, 2-s2.0-85015968146. PMC5359827 28320393

[29] Song N. , Xiong W. , Zhu J. , Zhu C. , Gu X. , and Yu Z. , Peptide-Based Strategies for Glioblastoma Therapy: From Small Molecular Drugs to Delivery Vehicles, Journal of Controlled Release. (2025) 385, 10.1016/j.jconrel.2025.114023. 40651601

[30] Jourdain M. A. and Eyer J. , Recent Advances in Liposomes and Peptide-Based Therapeutics for Glioblastoma Treatment, Journal of Controlled Release. (2024) 376, 732–752, 10.1016/j.jconrel.2024.10.037. 39437968

[31] King G. F. , Venoms as a Platform for Human Drugs: Translating Toxins Into Therapeutics, Expert Opinion on Biological Therapy. (2011) 11, no. 11, 1469–1484, 10.1517/14712598.2011.621940, 2-s2.0-80053645532. 21939428

[32] Freuville L. , Matthys C. , Quinton L. , and Gillet J. P. , Venom-Derived Peptides for Breaking Through the Glass Ceiling of Drug Development, Frontiers in Chemistry. (2024) 12, 10.3389/fchem.2024.1465459. PMC11468230 39398192

[33] Ghodeif S. K. , El-Fahla N. A. , Abdel-Rahman M. A. , and El-Shenawy N. S. , Arthropod Venom Peptides: Pioneering Nanotechnology in Cancer Treatment and Drug Delivery, Cancer Pathogenesis and Therapy, 2025. 10.1016/j.cpt.2025.03.005 PMC12800701 41541913

[34] Rathore A. S. , Kumar N. , Choudhury S. , Mehta N. K. , and Raghava G. P. S. , Prediction of Hemolytic Peptides and Their Hemolytic Concentration, Communications Biology. (2025) 8, no. 1, 10.1038/s42003-025-07615-w. PMC11794569 39905233

[35] Hasan M. M. , Schaduangrat N. , Basith S. , Lee G. , Shoombuatong W. , and Manavalan B. , HLPpred-Fuse: Improved and Robust Prediction of Hemolytic Peptide and Its Activity by Fusing Multiple Feature Representation, Bioinformatics. (2020) 36, no. 11, 3350–3356, 10.1093/bioinformatics/btaa160. 32145017

[36] Rathore A. S. , Choudhury S. , Arora A. , Tijare P. , and Raghava G. P. S. , ToxinPred 3.0: An Improved Method for Predicting the Toxicity of Peptides, Computers in Biology and Medicine. (2024) 179, 10.1016/j.compbiomed.2024.108926. 39038391

[37] Morozov V. , Rodrigues C. H. M. , and Ascher D. B. , CSM-Toxin: A Web-Server for Predicting Protein Toxicity, Pharmaceutics. (2023) 15, no. 2, 10.3390/pharmaceutics15020431. PMC9966851 36839752

[38] Pires D. E. V. , Blundell T. L. , and Ascher D. B. , pkCSM: Predicting Small-Molecule Pharmacokinetic and Toxicity Properties Using Graph-Based Signatures, Journal of Medicinal Chemistry. (2015) 58, no. 9, 4066–4072, 10.1021/acs.jmedchem.5b00104, 2-s2.0-84929377653. 25860834 PMC4434528

[39] Agrawal P. , Bhagat D. , Mahalwal M. , Sharma N. , and Raghava G. P. S. , AntiCP 2.0: An Updated Model for Predicting Anticancer Peptides, Briefings in Bioinformatics. (2021) 22, no. 3, 10.1093/bib/bbaa153. 32770192

[40] Thi Phan L. , Woo Park H. , Pitti T. , Madhavan T. , Jeon Y. J. , and Manavalan B. , MLACP 2.0: An Updated Machine Learning Tool for Anticancer Peptide Prediction, Computational and Structural Biotechnology Journal. (2022) 20, 4473–4480, 10.1016/j.csbj.2022.07.043. 36051870 PMC9421197

[41] Kumar P. , Saini V. , Gupta D. , Chawla P. A. , and Kumar A. , BBBper: A Machine Learning-Based Online Tool for Blood-Brain Barrier (BBB) Permeability Prediction, CNS and Neurological Disorders: Drug Targets. (2024) 24, 10.2174/0118715273328174241007060331. 39415574

[42] Shaker B. , Lee J. , Lee Y. et al., A Machine Learning-Based Quantitative Model (LogBB_Pred) to Predict the Blood–Brain Barrier Permeability (logBB Value) of Drug Compounds, Bioinformatics. (2023) 39, no. 10, 10.1093/bioinformatics/btad577. PMC10560102 37713469

[43] Giribaldi J. , Smith J. J. , and Schroeder C. I. , Recent Developments in Animal Venom Peptide Nanotherapeutics With Improved Selectivity for Cancer Cells, Biotechnology Advances. (2021) 50, 10.1016/j.biotechadv.2021.107769. 33989705

[44] Han Y. , Zhang M. , Lai R. , and Zhang Z. , Chemical Modifications to Increase the Therapeutic Potential of Antimicrobial Peptides, Peptides. (2021) 146, 10.1016/j.peptides.2021.170666. 34600037

[45] Vadevoo S. M. P. , Gurung S. , Lee H.-S. et al., Peptides as Multifunctional Players in Cancer Therapy, Experimental & Molecular Medicine. (2023) 55, no. 6, 1099–1109, 10.1038/s12276-023-01016-x. 37258584 PMC10318096

[46] Shahzadi S. K. , Karuvantevida N. , and Banerjee Y. , A Venomics Approach to the Identification and Characterization of Bioactive Peptides From Animal Venoms for Colorectal Cancer Therapy: Protocol for a Proof-of-Concept Study, JMIR Research Protocols. (2021) 10, no. 12, 10.2196/31128. PMC8734912 34932002

[47] Romano J. D. , Li H. , Napolitano T. et al., Discovering Venom-Derived Drug Candidates Using Differential Gene Expression, Toxins. (2023) 15, no. 7, 10.3390/toxins15070451. PMC10467105 37505720

[48] Catalog W. S. , World Spider Catalog, 2025, http://wsc.nmbe.ch.

[49] Wu T. , Wang M. , Wu W. et al., Spider Venom Peptides as Potential Drug Candidates Due to Their Anticancer and Antinociceptive Activities, Journal of Venomous Animals and Toxins Including Tropical Diseases. (2019) 25, 10.1590/1678-9199-jvatitd-14-63-18. PMC6551028 31210759

[50] Korolkova Y. , Mikov A. , Lobas A. et al., Venom-Gland Transcriptomics and Venom Proteomics of the Tibellus Oblongus Spider, Scientific Data. (2023) 10, no. 1, 10.1038/s41597-023-02703-0. PMC10665394 37993463

[51] Pineda S. S. , Undheim E. A. , Rupasinghe D. B. , Ikonomopoulou M. P. , and King G. F. , Spider Venomics: Implications for Drug Discovery, Future Medicinal Chemistry. (2014) 6, no. 15, 1699–1714, 10.4155/fmc.14.103, 2-s2.0-84911922472. 25406008

[52] You Y. , Yin W. , Tembrock L. R. et al., Transcriptome Sequencing of Wolf Spider Lycosa sp. (Araneae: Lycosidae) Venom Glands Provides Insights Into the Evolution and Diversity of Disulfide-Rich Toxins, Comparative Biochemistry and Physiology, Part D: Genomics and Proteomics. (2023) 48, 10.1016/j.cbd.2023.101145. 37748227

[53] Michálek O. , Walker A. A. , Šedo O. , Zdráhal Z. , King G. F. , and Pekár S. , Composition and Toxicity of Venom Produced by Araneophagous White-Tailed Spiders (Lamponidae: Lampona Sp.), Scientific Reports. (2022) 12, no. 1, 10.1038/s41598-022-24694-5. PMC9751281 36517485

[54] Rapôso C. , Scorpion and Spider Venoms in Cancer Treatment: State of the Art, Challenges, and Perspectives, Journal of Clinical and Translational Research. (2017) 3, no. 2, 233–249. 30873475 PMC6410669

[55] Bubien J. K. , Ji H. L. , Gillespie G. Y. et al., Cation Selectivity and Inhibition of Malignant Glioma Na+ Channels by Psalmotoxin 1, American Journal of Physiology: Cell Physiology. (2004) 287, no. 5, C1282–C1291, 10.1152/ajpcell.00077.2004, 2-s2.0-6044229552. 15253892

[56] Rooj A. K. , McNicholas C. M. , Bartoszewski R. , Bebok Z. , Benos D. J. , and Fuller C. M. , Glioma-Specific Cation Conductance Regulates Migration and Cell Cycle Progression, Journal of Biological Chemistry. (2012) 287, no. 6, 4053–4065, 10.1074/jbc.m111.311688, 2-s2.0-84856740568. 22130665 PMC3281704

[57] Nicoletti N. F. , Erig T. C. , Zanin R. F. et al., Pre-Clinical Evaluation of Voltage-Gated Calcium Channel Blockers Derived From the Spider P. Nigriventer in Glioma Progression, Toxicon. (2017) 129, 58–67, 10.1016/j.toxicon.2017.02.001, 2-s2.0-85013766266. 28202361

[58] Abdel-Salam Mal C.-T. J. , Gomes K. S. , Teixeira-Carvalho A. et al., The Synthetic Peptide LyeTxI-b Derived From Lycosa Erythrognatha Spider Venom is Cytotoxic to U-87 MG Glioblastoma Cells, Amino Acids. (2019) 51, no. 3, 433–449, 10.1007/s00726-018-2678-4, 2-s2.0-85056733482. 30449002

[59] Lyons S. A. , O’Neal J. , and Sontheimer H. , Chlorotoxin, A Scorpion-Derived Peptide, Specifically Binds to Gliomas and Tumors of Neuroectodermal Origin, Glia. (2002) 39, no. 2, 162–173, 10.1002/glia.10083, 2-s2.0-0035983279. 12112367

[60] Fu Y. J. , An N. , Chan K. G. , Wu Y. B. , Zheng S. H. , and Liang A. H. , A Model of BmK CT in Inhibiting Glioma Cell Migration Via Matrix Metalloproteinase-2 From Experimental and Molecular Dynamics Simulation Study, Biotechnology Letters. (2011) 33, no. 7, 1309–1317, 10.1007/s10529-011-0587-7, 2-s2.0-79958773007. 21424168

[61] Rjeibi I. , Mabrouk K. , Mosrati H. et al., Purification, Synthesis and Characterization of AaCtx, the First Chlorotoxin-Like Peptide From Androctonus Australis Scorpion Venom, Peptides. (2011) 32, no. 4, 656–663, 10.1016/j.peptides.2011.01.015, 2-s2.0-79952816041. 21262299

[62] Caliskan F. , Ergene E. , Sogut I. et al., Biological Assays on the Effects of Acra3 Peptide From Turkish Scorpion Androctonus Crassicauda Venom on a Mouse Brain Tumor Cell Line (BC3H1) and Production of Specific Monoclonal Antibodies, Toxicon. (2013) 76, 350–361, 10.1016/j.toxicon.2013.09.009, 2-s2.0-84889101323. 24055552

[63] Othman H. , Wieninger S. A. , ElAyeb M. , Nilges M. , and Srairi-Abid N. , In Silico Prediction of the Molecular Basis of ClTx and AaCTx Interaction With Matrix Metalloproteinase-2 (MMP-2) to Inhibit Glioma Cell Invasion, Journal of Biomolecular Structure and Dynamics. (2017) 35, no. 13, 2815–2829, 10.1080/07391102.2016.1231633, 2-s2.0-84988917767. 27678152

[64] Aissaoui D. , Mlayah-Bellalouna S. , Jebali J. et al., Functional Role of Kv1.1 and Kv1.3 Channels in the Neoplastic Progression Steps of Three Cancer Cell Lines, Elucidated by Scorpion Peptides, International Journal of Biological Macromolecules. (2018) 111, 1146–1155, 10.1016/j.ijbiomac.2018.01.144, 2-s2.0-85041389645. 29415410

[65] Khamessi O. , Ben Mabrouk H. , Othman H. et al., RK, The First Scorpion Peptide With Dual Disintegrin Activity on α(1)β(1) and α(v)β(3) Integrins, International Journal of Biological Macromolecules. (2018) 120, 1777–1788, 10.1016/j.ijbiomac.2018.09.180, 2-s2.0-85054298847. 30287364

[66] Khamessi O. , Ben Mabrouk H. , ElFessi-Magouri R. , and Kharrat R. , RK1, The First Very Short Peptide From Buthus Occitanus Tunetanus Inhibits Tumor Cell Migration, Proliferation and Angiogenesis, Biochemical and Biophysical Research Communications. (2018) 499, no. 1, 1–7, 10.1016/j.bbrc.2018.01.133, 2-s2.0-85044603825. 29366787

[67] Aissaoui-Zid D. , Saada M. C. , Moslah W. et al., AaTs-1: A Tetrapeptide From Androctonus australis Scorpion Venom, Inhibiting U87 Glioblastoma Cells Proliferation by p53 and FPRL-1 Up-Regulations, Molecules. (2021) 26, no. 24, 10.3390/molecules26247610. PMC8704564 34946686

[68] Mlayah-Bellalouna S. , Aissaoui-Zid D. , Chantome A. et al., Insights Into the Mechanisms Governing P01 Scorpion Toxin Effect Against U87 Glioblastoma Cells Oncogenesis, Frontiers in Pharmacology. (2023) 14, 10.3389/fphar.2023.1203247. PMC10326281 37426811

[69] Wang K. , Nguyen T. , Gao Y. et al., Androcin 18-1, A Novel Scorpion-Venom Peptide, Shows a Potent Antitumor Activity Against Human U87 Cells Via Inducing Mitochondrial Dysfunction, Insect Biochemistry and Molecular Biology. (2024) 170, 10.1016/j.ibmb.2024.104137. 38759703

[70] Reis P. V. M. , Boff D. , Verly R. M. et al., LyeTxI-b, A Synthetic Peptide Derived From Lycosa Erythrognatha Spider Venom, Shows Potent Antibiotic Activity In Vitro and In Vivo, Frontiers in Microbiology. (2018) 9, 10.3389/fmicb.2018.00667, 2-s2.0-85045036663. PMC5897548 29681894

[71] Peigneur S. , de Lima M. E. , and Tytgat J. , Phoneutria Nigriventer Venom: A Pharmacological Treasure, Toxicon. (2018) 151, 96–110, 10.1016/j.toxicon.2018.07.008, 2-s2.0-85049724029. 30003916

[72] Soroceanu L. , Gillespie Y. , Khazaeli M. B. , and Sontheimer H. , Use of Chlorotoxin for Targeting of Primary Brain Tumors, Cancer Research. (1998) 58, no. 21, 4871–4879. 9809993

[73] Ghosh A. , Roy R. , Nandi M. , and Mukhopadhyay A. , Scorpion Venom-Toxins That Aid in Drug Development: A Review, International Journal of Peptide Research and Therapeutics. (2019) 25, no. 1, 27–37, 10.1007/s10989-018-9721-x, 2-s2.0-85048039970. 32214927 PMC7088386

[74] Dueñas-Cuellar R. A. , Santana C. J. C. , Magalhães A. C. M. , Pires O. R.Jr., Fontes W. , and Castro M. S. , Scorpion Toxins and Ion Channels: Potential Applications in Cancer Therapy, Toxins. (2020) 12, no. 5, 10.3390/toxins12050326. PMC7290751 32429050

[75] Purali N. and Yagcioglu S. , Lidocaine Diminishes Arrhythmia by Leiurus Quinquestriatus Quinquestriatus Venom in Rats, Fundamental & clinical Pharmacology. (2002) 16, no. 3, 227–235, 10.1046/j.1472-8206.2002.00071.x, 2-s2.0-0036424458. 12165070

[76] Chaïr-Yousfi I. , Laraba-Djebari F. , and Hammoudi-Triki D. , Androctonus australis Hector Venom Contributes to the Interaction Between Neuropeptides and Mast Cells in Pulmonary Hyperresponsiveness, International Immunopharmacology. (2015) 25, no. 1, 19–29, 10.1016/j.intimp.2015.01.008, 2-s2.0-84921532550. 25601496

[77] ElFessi-Magouri R. , Peigneur S. , Khamessi O. et al., Kbot55, Purified From Buthus Occitanus Tunetanus Venom, Represents the First Member of a Novel α-KTx Subfamily, Peptides. (2016) 80, 4–8, 10.1016/j.peptides.2015.05.015, 2-s2.0-84933055826. 26079392

[78] Perez-Riverol A. , Lasa A. M. , dos Santos-Pinto J. R. A. , and Palma M. S. , Insect Venom Phospholipases A1 and A2: Roles in the Envenoming Process and Allergy, Insect Biochemistry and Molecular Biology. (2019) 105, 10–24, 10.1016/j.ibmb.2018.12.011, 2-s2.0-85059447706. 30582958

[79] Laurindo L. F. , de Lima E. P. , Laurindo L. F. et al., The Therapeutic Potential of Bee Venom-Derived Apamin and Melittin Conjugates in Cancer Treatment: A Systematic Review, Pharmacological Research. (2024) 209, 10.1016/j.phrs.2024.107430. 39332751

[80] da Silva A. M. B. , Silva-Gonçalves L. C. , Oliveira F. A. , and Arcisio-Miranda M. , Pro-Necrotic Activity of Cationic Mastoparan Peptides in Human Glioblastoma Multiforme Cells Via Membranolytic Action, Molecular Neurobiology. (2018) 55, no. 7, 5490–5504, 10.1007/s12035-017-0782-1, 2-s2.0-85030160162. 28965321

[81] Lebel A. A. , Kisembo M. V. , Soucy M. N. , Hébert M. P. A. , Morin P. J. , and Boudreau L. H. , Molecular Characterization of the Anticancer Properties Associated With Bee Venom and Its Components in Glioblastoma Multiforme, Chemico-Biological Interactions. (2021) 347, 10.1016/j.cbi.2021.109622. 34375656

[82] Ertilav K. and Nazıroğlu M. , Honey Bee Venom Melittin Increases the Oxidant Activity of Cisplatin and Kills Human Glioblastoma Cells by Stimulating the TRPM2 Channel, Toxicon. (2023) 222, 10.1016/j.toxicon.2022.106993. 36528210

[83] Abd El-Wahed A. , Yosri N. , Sakr H. H. et al., Wasp Venom Biochemical Components and Their Potential in Biological Applications and Nanotechnological Interventions, Toxins. (2021) 13, no. 3, 10.3390/toxins13030206. PMC8000949 33809401

[84] Oršolić N. , Bee Venom in Cancer Therapy, Cancer and Metastasis Reviews. (2012) 31, no. 1-2, 173–194, 10.1007/s10555-011-9339-3, 2-s2.0-84862337185. 22109081

[85] Münstedt K. and Männle H. , Bee Products and Their Role in Cancer Prevention and Treatment, Complementary Therapies in Medicine. (2020) 51, 10.1016/j.ctim.2020.102390. 32507447

[86] Wehbe R. , Frangieh J. , Rima M. , El Obeid D. , Sabatier J. M. , and Fajloun Z. , Bee Venom: Overview of Main Compounds and Bioactivities for Therapeutic Interests, Molecules. (2019) 24, no. 16, 10.3390/molecules24162997, 2-s2.0-85070760932. PMC6720840 31430861

[87] Ali A. M. and Kunugi H. , Royal Jelly as an Intelligent Anti-Aging Agent-A Focus on Cognitive Aging and Alzheimer’s Disease: A Review, Antioxidants. (2020) 9, no. 10, 10.3390/antiox9100937. PMC7601550 33003559

[88] Olas B. , Bee Products as Interesting Natural Agents for the Prevention and Treatment of Common Cardiovascular Diseases, Nutrients. (2022) 14, no. 11, 10.3390/nu14112267. PMC9182958 35684067

[89] Nowak A. , Szczuka D. , Górczyńska A. , Motyl I. , and Kręgiel D. , Characterization of *Apis mellifera* Gastrointestinal Microbiota and Lactic Acid Bacteria for Honeybee Protection-A Review, Cells. (2021) 10, no. 3, 10.3390/cells10030701. PMC8004194 33809924

[90] Zheng J. , Lee H. L. , Ham Y. W. , Song H. S. , Song M. J. , and Hong J. T. , Anti-Cancer Effect of Bee Venom on Colon Cancer Cell Growth by Activation of Death Receptors and Inhibition of Nuclear Factor Kappa B, Oncotarget. (2015) 6, no. 42, 44437–44451, 10.18632/oncotarget.6295, 2-s2.0-84953394436. 26561202 PMC4792567

[91] Małek A. , Kocot J. , Mitrowska K. , Posyniak A. , and Kurzepa J. , Bee Venom Effect on Glioblastoma Cells Viability and Gelatinase Secretion, Frontiers in Neuroscience. (2022) 16, 10.3389/fnins.2022.792970. PMC8873382 35221898

[92] Defendini M. L. , Juin M. , and Granier C. , Apamin Binding Sites in Human C6 Cell Line and Their Accessibility to Monoclonal Antibodies, NeuroReport. (1990) 1, no. 3-4, 229–231, 10.1097/00001756-199011000-00014, 2-s2.0-0025542908. 1966607

[93] Waheed H. , Moin S. F. , and Choudhary M. I. , Snake Venom: From Deadly Toxins to Life-Saving Therapeutics, Current Medicinal Chemistry. (2017) 24, no. 17, 1874–1891, 10.2174/0929867324666170605091546, 2-s2.0-85026754345. 28578650

[94] Almeida J. R. , Gomes A. , Mendes B. et al., Unlocking the Potential of Snake Venom-Based Molecules Against the Malaria, Chagas Disease, and Leishmaniasis Triad, International Journal of Biological Macromolecules. (2023) 242, 10.1016/j.ijbiomac.2023.124745. 37150376

[95] Teodoro A. , Gonçalves F. J. M. , Oliveira H. , and Marques S. , Venom of Viperidae: A Perspective of Its Antibacterial and Antitumor Potential, Current Drug Targets. (2022) 23, no. 2, 126–144, 10.2174/1389450122666210811164517. 35139779

[96] Pérez-Peinado C. , Defaus S. , and Andreu D. , Hitchhiking With Nature: Snake Venom Peptides to Fight Cancer and Superbugs, Toxins. (2020) 12, no. 4, 10.3390/toxins12040255. PMC7232197 32326531

[97] Schmitmeier S. , Markland F. S. , Ritter M. R. , Sawcer D. E. , and Chen T. C. , Functional Effect of Contortrostatin, A Snake Venom Disintegrin, on Human Glioma Cell Invasion In Vitro, Cell Communication and Adhesion. (2003) 10, no. 1, 1–16, 10.1080/15419060302062, 2-s2.0-0042029325. 12881036

[98] Pyrko P. , Wang W. , Markland F. S. et al., The Role of Contortrostatin, A Snake Venom Disintegrin, in the Inhibition of Tumor Progression and Prolongation of Survival in a Rodent Glioma Model, Journal of Neurosurgery. (2005) 103, no. 3, 526–537, 10.3171/jns.2005.103.3.0526, 2-s2.0-27244451494. 16235686

[99] Morjen M. , Kallech-Ziri O. , Bazaa A. et al., PIVL, A New Serine Protease Inhibitor From *Macrovipera lebetina* Transmediterranea Venom, Impairs Motility of Human Glioblastoma Cells, Matrix Biology. (2013) 32, no. 1, 52–62, 10.1016/j.matbio.2012.11.015, 2-s2.0-84873250923. 23262217

[100] Swenson S. , Minea R. O. , Tuan C. D. , Thein T. Z. , Chen T. C. , and Markland F. S. , A Novel Venom-Derived Peptide for Brachytherapy of Glioblastoma: Preclinical Studies in Mice, Molecules. (2018) 23, no. 11, 10.3390/molecules23112918, 2-s2.0-85056374695. PMC6278533 30413113

[101] González García M. C. , Radix C. , Villard C. et al., Myotoxin-3 From the Pacific Rattlesnake Crotalus Oreganus Oreganus Venom is a New Microtubule-Targeting Agent, Molecules. (2022) 27, no. 23, 10.3390/molecules27238241. PMC9739105 36500334

[102] Dos Santos N. F. T. , Imberg A. S. , Mariano D. O. C. et al., β-micrustoxin (Mlx-9), a PLA(2) From *Micrurus lemniscatus* Snake Venom: Biochemical Characterization and Anti-Proliferative Effect Mediated by p53, Journal of Venomous Animals and Toxins including Tropical Diseases. (2022) 28, 10.1590/1678-9199-jvatitd-2021-0094. PMC9008913 35432496

[103] Galicka A. , Szoka Ł. , Radziejewska I. , and Marcinkiewicz C. , Effect of Dimeric Disintegrins Isolated From Vipera Lebetina Obtusa Venom on Glioblastoma Cellular Responses, Cancers (Basel). (2023) 15, no. 19, 10.3390/cancers15194805. PMC10572073 37835499

[104] Hamza L. , Girardi T. , Castelli S. et al., Isolation and Characterization of a Myotoxin From the Venom of *Macrovipera lebetina* Transmediterranea, Toxicon. (2010) 56, no. 3, 381–390, 10.1016/j.toxicon.2010.04.001, 2-s2.0-77954143769. 20398688

[105] Bocian A. , Urbanik M. , Hus K. et al., Proteomic Analyses of *Agkistrodon contortrix* Contortrix Venom Using 2D Electrophoresis and MS Techniques, Toxins. (2016) 8, no. 12, 10.3390/toxins8120372, 2-s2.0-85006729178. PMC5198566 27983581

[106] Robles-Loaiza A. A. , Pinos-Tamayo E. A. , Mendes B. et al., Traditional and Computational Screening of Non-Toxic Peptides and Approaches to Improving Selectivity, Pharmaceuticals. (2022) 15, no. 3, 10.3390/ph15030323. PMC8953747 35337121

[107] Wu X. , Lin H. , Bai R. , and Duan H. , Deep Learning for Advancing Peptide Drug Development: Tools and Methods in Structure Prediction and Design, European Journal of Medicinal Chemistry. (2024) 268, 10.1016/j.ejmech.2024.116262. 38387334

[108] Jamialahmadi H. , Khalili-Tanha G. , Nazari E. , and Rezaei-Tavirani M. , Artificial Intelligence and Bioinformatics: A Journey From Traditional Techniques to Smart Approaches, Gastroenterol Hepatol Bed Bench. (2024) 17, no. 3, 241–252, 10.22037/ghfbb.v17i3.2977. 39308539 PMC11413381

[109] Siddiqui B. , Yadav C. S. , Akil M. et al., Artificial Intelligence in Computer-Aided Drug Design (CADD) Tools for the Finding of Potent Biologically Active Small Molecules: Traditional to Modern Approach, Combinatorial Chemistry & High Throughput Screening. (2025) 28, 10.2174/0113862073334062241015043343. 39819404

[110] Kumar N. and Srivastava R. , Deep Learning in Structural Bioinformatics: Current Applications and Future Perspectives, Briefings in Bioinformatics. (2024) 25, no. 3, 10.1093/bib/bbae042. PMC11066934 38701422

[111] Alam S. , Israr J. , and Kumar A. , Singh V. and Kumar A. , Artificial Intelligence and Machine Learning in Bioinformatics, Advances in Bioinformatics, 2024, Springer Nature Singapore, 321–345.

[112] Obaido G. , Mienye I. D. , Egbelowo O. F. et al., Supervised Machine Learning in Drug Discovery and Development: Algorithms, Applications, Challenges, and Prospects, Machine Learning With Applications. (2024) 17, 10.1016/j.mlwa.2024.100576.

[113] Gupta R. , Srivastava D. , Sahu M. , Tiwari S. , Ambasta R. K. , and Kumar P. , Artificial Intelligence to Deep Learning: Machine Intelligence Approach for Drug Discovery, Molecular Diversity. (2021) 25, no. 3, 1315–1360, 10.1007/s11030-021-10217-3. 33844136 PMC8040371

[114] Yaseen A. , Gull S. , Akhtar N. , Amin I. , and Minhas F. , HemoNet: Predicting Hemolytic Activity of Peptides With Integrated Feature Learning, Journal of Bioinformatics and Computational Biology. (2021) 19, no. 05, 10.1142/s0219720021500219. 34353244

[115] Salam A. , Ullah F. , Amin F. et al., Efficient Prediction of Anticancer Peptides Through Deep Learning, PeerJ Computer Science. (2024) 10, 10.7717/peerj-cs.2171. PMC11323142 39145253

[116] Nasiri F. , Atanaki F. F. , Behrouzi S. , Kavousi K. , and Bagheri M. , CpACpP: In Silico Cell-Penetrating Anticancer Peptide Prediction Using a Novel Bioinformatics Framework, ACS Omega. (2021) 6, no. 30, 19846–19859, 10.1021/acsomega.1c02569. 34368571 PMC8340416

[117] Feijoo-Coronel M. L. , Mendes B. , Ramírez D. et al., Antibacterial and Antiviral Properties of Chenopodin-Derived Synthetic Peptides, Antibiotics. (2024) 13, no. 1, 10.3390/antibiotics13010078. PMC10812719 38247637

[118] Mendes B. , Edwards-Gayle C. , and Barrett G. , Peptide Lipidation and Shortening Optimises Antibacterial, Antibiofilm and Membranolytic Actions of an Amphiphilic polylysine-Polyphenyalanine Octapeptide, Current Research in Biotechnology. (2024) 8, 10.1016/j.crbiot.2024.100240.

[119] Peña-Carrillo M. S. , Pinos-Tamayo E. A. , Mendes B. et al., Dissection of Phospholipases A2 Reveals Multifaceted Peptides Targeting Cancer Cells, Leishmania and Bacteria, Bioorganic Chemistry. (2021) 114, 10.1016/j.bioorg.2021.105041. 34130109

[120] Zhong L. , Li Y. , Xiong L. et al., Small Molecules in Targeted Cancer Therapy: Advances, Challenges, and Future Perspectives, Signal Transduction and Targeted Therapy. (2021) 6, no. 1, 10.1038/s41392-021-00572-w. PMC8165101 34054126

[121] Setiawati A. , Candrasari D. S. , Setyajati F. D. E. , Prasetyo V. K. , Setyaningsih D. , and Hartini Y. S. , Anticancer Drug Screening of Natural Products: In Vitro: Cytotoxicity Assays, Techniques, and Challenges, Asian Pacific Journal of Tropical Biomedicine. (2022) 12, no. 7, 279–289, 10.4103/2221-1691.350176.

[122] Jain A. K. , Singh D. , Dubey K. , Maurya R. , Mittal S. , and Pandey A. K. , Dhawan A. and Kwon S. , Chapter 3-Models and Methods for In Vitro Toxicity, Vitro Toxicology, 2018, Academic Press, 45–65.

[123] von Petersdorff-Campen K. and Schmid Daners M. , Hemolysis Testing In Vitro: A Review of Challenges and Potential Improvements, ASAIO Journal. (2022) 68, no. 1, 3–13, 10.1097/mat.0000000000001454. 33989208

[124] Sæbø I. P. , Bjørås M. , Franzyk H. , Helgesen E. , and Booth J. A. , Optimization of the Hemolysis Assay for the Assessment of Cytotoxicity, International Journal of Molecular Sciences. (2023) 24, no. 3, 10.3390/ijms24032914. PMC9917735 36769243

[125] Gabaj N. N. , Miler M. , Vrtaric A. et al., Comparison of Three Different Protocols for Obtaining Hemolysis, Clinical Chemistry and Laboratory Medicine. (2022) 60, no. 5, 714–725, 10.1515/cclm-2021-1227. 35212494

[126] Ridder M. J. , Daly S. M. , Hall P. R. , and Bose J. L. , Rice K. C. , Quantitative Hemolysis Assays, Staphylococcus aureus: Methods and Protocols, 2021, Springer US, 25–30. 10.1007/978-1-0716-1550-8_4 34264457

[127] Greco I. , Molchanova N. , Holmedal E. et al., Correlation Between Hemolytic Activity, Cytotoxicity and Systemic In Vivo Toxicity of Synthetic Antimicrobial Peptides, Scientific Reports. (2020) 10, no. 1, 10.1038/s41598-020-69995-9. PMC7414031 32764602

[128] Chiangjong W. , Chutipongtanate S. , and Hongeng S. , Anticancer Peptide: Physicochemical Property, Functional Aspect and Trend in Clinical Application (Review), International Journal of Oncology. (2020) 57, no. 3, 678–696, 10.3892/ijo.2020.5099. 32705178 PMC7384845

[129] Huang Y. B. , Wang X. F. , Wang H. Y. , Liu Y. , and Chen Y. , Studies on Mechanism of Action of Anticancer Peptides by Modulation of Hydrophobicity Within a Defined Structural Framework, Molecular Cancer Therapeutics. (2011) 10, no. 3, 416–426, 10.1158/1535-7163.mct-10-0811, 2-s2.0-79955748813. 21252288

[130] Glukhov E. , Burrows L. L. , and Deber C. M. , Membrane Interactions of Designed Cationic Antimicrobial Peptides: The Two Thresholds, Biopolymers. (2008) 89, no. 5, 360–371, 10.1002/bip.20917, 2-s2.0-47749118037. 18186149

[131] Charoenkwan P. , Chiangjong W. , Lee V. S. , Nantasenamat C. , Hasan M. M. , and Shoombuatong W. , Improved Prediction and Characterization of Anticancer Activities of Peptides Using a Novel Flexible Scoring Card Method, Scientific Reports. (2021) 11, no. 1, 10.1038/s41598-021-82513-9. PMC7862624 33542286

[132] Hadianamrei R. , Tomeh M. A. , Brown S. , Wang J. , and Zhao X. , Rationally Designed Short Cationic α-Helical Peptides With Selective Anticancer Activity, Journal of Colloid and Interface Science. (2022) 607, 488–501, 10.1016/j.jcis.2021.08.200. 34509120

[133] Guo F. , Zhang Y. , Dong W. , Guan Y. , and Shang D. , Effect of Hydrophobicity on Distinct Anticancer Mechanism of Antimicrobial Peptide Chensinin-1b and Its Lipoanalog PA-C1b in Breast Cancer Cells, The International Journal of Biochemistry & Cell Biology. (2022) 143, 10.1016/j.biocel.2022.106156. 34999227

[134] Sun B. , Li R. , Ji N. et al., Brain-Targeting Drug Delivery Systems: The State of the Art in Treatment of Glioblastoma, Materials Today Bio. (2025) 30, 10.1016/j.mtbio.2025.101443. PMC11759563 39866779

[135] Banks W. A. , Rhea E. M. , Reed M. J. , and Erickson M. A. , The Penetration of Therapeutics Across the Blood-Brain Barrier: Classic Case Studies and Clinical Implications, Cell Reports Medicine. (2024) 5, no. 11, 10.1016/j.xcrm.2024.101760. PMC11604479 39383873

[136] Noorani I. and de la Rosa J. , Breaking Barriers for Glioblastoma With a Path to Enhanced Drug Delivery, Nature Communications. (2023) 14, no. 1, 10.1038/s41467-023-41694-9. PMC10517119 37737212

[137] Damani M. , Nilawar N. , Momin M. , Ningthoujham R. S. , and Khan T. , Nanoparticles Assisted Drug Delivery for Effective Management of Glioblastoma, Nanotechnology. (2025) 7, 10.1016/j.nxnano.2025.100137.

